# Instruments for assessing the impacts of oil spills: an integrated approach to health, the environment and the socioeconomic profile of exposed areas

**DOI:** 10.1590/0102-311XEN228723

**Published:** 2025-03-31

**Authors:** Loic Hernandez do Amaral e Aragão, Maria Juliana Ferreira dos Santos, Aline do Monte Gurgel, Mariana Olivia Santana dos Santos, Mariana Maciel Nepomuceno, Idê Gomes Dantas Gurgel

**Affiliations:** 1 Instituto Aggeu Magalhães, Fundação Oswaldo Cruz, Recife, Brasil.; 2 Faculdade de Ciências da Saúde, Universidade Federal do Rio Grande do Norte, Santa Cruz, Brasil.; 3 Centro Acadêmico do Agreste, Universidade Federal de Pernambuco, Caruaru, Brasil.; 4 Faculdade Pernambucana de Saúde, Recife, Brasil.

**Keywords:** Petroleum Pollution, Surveys and Questionnaires, Disaster Evaluation, Contaminación por Petróleo, Encuestas y Cuestionarios, Evaluación de Desastres

## Abstract

Advances in the oil industry have been associated with major disasters involving oil spills in offshore fields, negatively impacting life and the environment. We considered the importance of monitoring and evaluating these events, using various instruments, according to three research axes: health; the environment; and the socioeconomic situation of exposed populations. Thus, the objective was to survey, through a scoping review, scientific evidence involving the application of these instruments to assess the impacts of oil spills. Different databases and languages were used to search for the works. The data were reviewed by a pair of researchers, who carried out the qualitative evaluation. For synthesis of the results, we considered 45 studies distributed among observational studies with no control group, cohort studies with control group, and cross-sectional studies, with a predominance of studies focused on the health axis (n = 39; 86.66%) and with interview method (n = 29; 64.44%). We found 75 records of instruments used, with Likert-type scales, combined scales and free response patterns. In addition, there was a gap in studies on the environmental and socioeconomic axes, especially in an integrated manner. Finally, we considered the importance of new research including essential characteristics of the instruments (consistency, reliability, faithfulness, cross-cultural adaptations) for the possibility of building multidimensional matrices to monitor disasters caused by human action, facilitating decision-making in the formulation of government policies and actions.

## Abstract (es)

Los avances en la industria petrolera están asociados a grandes desastres que involucran derrames en campos marítimos, con repercusiones negativas en la vida y en el medioambiente. Es importante monitorear y evaluar estos eventos a partir de diversas herramientas divididas en tres ejes de investigación: la salud; el medioambiente; y la situación socioeconómica de las poblaciones expuestas. A partir de una revisión de alcance, este estudio pretende mapear la evidencia científica sobre la aplicación de estas herramientas acerca de los impactos de los derrames de petróleo. Se utilizaron diferentes bases de datos e idiomas en la búsqueda de estudios. Los datos fueron revisados por dos investigadores, quienes realizaron la evaluación cualitativa. Para la síntesis de los resultados se consideraron 45 estudios distribuidos entre observacionales sin grupo control, de cohorte con grupo control y transversales, con predominio en el eje salud (n = 39; 86,66%) y como método la entrevista (n = 29; 64,44%). Se identificaron 75 registros de herramientas utilizadas, con escalas Likert, escalas combinadas y patrones de respuesta libre. Además, se observó la falta de estudios centrados en los ejes ambiental y socioeconómico, sobre todo de manera integrada. Por último, se destaca la importancia de que futuras investigaciones incluyan características esenciales de los instrumentos (consistencia, confiabilidad, fiabilidad, adaptaciones transculturales) para que se construyan matrices multidimensionales de monitoreo de desastres causados por la acción humana, facilitando la toma de decisiones en la elaboración de políticas y acciones gubernamentales.

## Introduction

Historically, industrial development and increasing demand for energy have driven global economic growth. In this context, the oil industry - which originated in the 19th century United States - was prominent as one of the world’s main energy sources ^1^ ^,^ ^2^ ^,^ ^3^. In addition, the economic growth of several nations has become heavily dependent on fossil fuels, such as oil, natural gas and coal, which directly drive the global economy and international trade ^4^.

The main oil reserves are in deep offshore fields, whose exploration requires constant technological advances. These advances are essential for extraction, transportation, and refining, but they also face geological, climatic, and operational challenges, which, when not adequately overcome, can result in major human, material, and environmental damage ^3^ ^,^ ^5^ ^,^ ^6^ ^,^ ^7^. Thus, it is important to emphasize that oil exploration is among the most polluting human activities, as it generates huge volumes of solid waste and harmful gases that degrade the environment and affect human health ^8^.

Accordingly, safety along the production chain is crucial to avoid major accidents, such as oil spills, which cause serious environmental damage and risks to the health of living beings, which become vulnerable to the increased incidence of cancer and neurological and psychological, integumentary, hepatic, cardiovascular and respiratory diseases ^9^ ^,^ ^10^.

Consistently, throughout history, several disasters involving oil have demonstrated the devastating impact of these occurrences. The first major case recorded was the Sinclair Petrolore (1960) disaster, followed by others such as Torrey Canyon (1967), Exxon Valdez (1989), Hebei Spirit (2007) and, more recently, Deepwater Horizon (2010) ^11^ ^,^ ^12^ ^,^ ^13^ ^,^ ^14^ ^,^ ^15^ ^,^ ^16^ ^,^ ^17^ ^,^ ^18^ ^,^ ^19^. Each of these disasters had severe consequences, both for the affected ecosystems and local populations, exposing them to serious health problems and economic losses.

In Brazil, the 2019 disaster showed the severity of oil spills. The oil slicks that appeared on the Northeastern coast affected more than 3,400km of coastline, directly impacting the ecosystem, the local economy and public health. In addition, the spill continues to pose environmental challenges, as oil fragments continue to appear on the beaches and the source of the problem has not been identified ^20^ ^,^ ^21^.

In the face of these disasters, the use of assessment and monitoring instruments has been fundamental to deal with the consequent impacts. Such instruments - including scales, questionnaires and checklists - are used to measure the consequences of spills and support decision-making in emergency situations ^22^ ^,^ ^23^. However, appropriately choosing these instruments is vital, as the diversity of involved variables can directly influence the reliability of the results ^24^.

Considering the gap in studies correlating the application of these instruments in contexts of oil spills, it is assumed, therefore, that, by identifying them, the monitoring, response, and intervention on the situation become more efficient and effective, enabling a better management of actions in different approaches, whether they are geared toward aspects of healthcare, environmental protection and recovery, and socioeconomic repairs.

Thus, the present study aims to survey scientific evidence related to the application of these instruments to assess and monitor the impacts of oil spills on the health, environment and socioeconomic profile of affected areas.

## Method

This is a scoping review, used to map a gap in a field of research of interest, being considered a preliminary stage to a systematic review with methodological rigor and reproducibility, in terms of nature, characteristics and volume ^25^ ^,^ ^26^ ^,^ ^27^.

For this approach, we adopted the international guide called *Preferred Reporting Items for Systematic Reviews and Meta-Analyses Extension for Scoping Reviews* (PRISMA-ScR) and the recommendation proposed by the Joanna Briggs Institute that is widely disseminated and adopted by several studies that use the scope review methodology in six stages: (i) definition of the question; (ii) identification of studies; (iii) selection of studies based on inclusion and exclusion criteria; (iv) data extraction; (v) organization of results; and (vi) dissemination of research reports ^28^ ^,^ ^29^ ^,^ ^30^.

This study was registered in the Open Science Framework (https://osf.io/y792v/) for storage and transparency regarding the protocol used. In addition, regarding the guiding question, the Population-Concept-Context (PCC) method was used to identify key topics, as follows: how are the instruments for measuring and monitoring impacts of oil spills used to assess the health, environment and socioeconomic profile of exposed populations and areas?

For the purposes of this study, the following databases were used: MEDLINE via PubMed; Cochrane Library; Virtual Health Library (VHL); Portal de Periódicos CAPES; Scopus; Web of Science; Embase; and SciELO. All databases present the scope of studies that meet the objective and the guiding question presented. In addition, we did not consider sources of gray literature, which were not formally published.

For the development of this study, the bibliographic search considered the relation of terms pertinent to the central object of the study, with boolean operators AND and OR, filtering the results in the different databases. The terms used and the search strategy were guided by the *Medical Subject Headings* (MeSH) - *petroleum pollution*, *surveys and questionnaires* and their respective entry terms, as shown in Box 1.

**Box 1:** Search strategies applied by database using AND and OR operators.

DATABASES	SEARCH STRATEGIES
PubMed	((((((((((((Petroleum Pollution[Title/Abstract]) OR (Petroleum Pollutions[Title/Abstract])) OR (Pollution, Petroleum[Title/Abstract])) OR (Pollutions, Petroleum[Title/Abstract])) OR (Oil Spills[Title/Abstract])) OR (Oil Spill[Title/Abstract])) OR (Spill, Oil[Title/Abstract])) OR (Spills, Oil[Title/Abstract])) OR (Oil Pollution[Title/Abstract])) OR (Oil Pollutions[Title/Abstract])) OR (Pollution, Oil[Title/Abstract])) OR (Pollutions, Oil[Title/Abstract])) AND ((((((((((((((((((((((((((((((((Surveys[Title/Abstract] AND Questionnaires[Title/Abstract]) OR (Questionnaires[Title/Abstract] AND Surveys[Title/Abstract])) OR (Survey Methods[Title/Abstract])) OR (Methods, Survey[Title/Abstract])) OR (Survey Method[Title/Abstract])) OR (Methodology, Survey[Title/Abstract])) OR (Survey Methodology[Title/Abstract])) OR (Community Surveys[Title/Abstract])) OR (Survey[Title/Abstract])) OR (Surveys[Title/Abstract])) OR (Repeated Rounds of Survey[Title/Abstract])) OR (Surveys, Community[Title/Abstract])) OR (Survey, Community[Title/Abstract])) OR (Respondent[Title/Abstract])) OR (Respondents[Title/Abstract])) OR (Surveys, Baseline[Title/Abstract])) OR (Survey, Baseline[Title/Abstract])) OR (Baseline Surveys[Title/Abstract])) OR (Baseline Survey[Title/Abstract])) OR (Questionnaire Designs[Title/Abstract])) OR (Designs, Questionnaire[Title/Abstract])) OR (Design, Questionnaire[Title/Abstract])) OR (Questionnaire Design[Title/Abstract])) OR (Nonrespondent[Title/Abstract])) OR (Nonrespondents[Title/Abstract])) OR (Questionnaire[Title/Abstract])) OR (Questionnaires[Title/Abstract])) OR (Techniques, Randomized Response[Title/Abstract])) OR (Response Techniques, Randomized[Title/Abstract])) OR (Response Technique, Randomized[Title/Abstract])) OR (Randomized Response Techniques[Title/Abstract])) OR (Randomized Response Technique[Title/Abstract]))
Cochrane Library	(Petroleum Pollution) OR (Spills, Oil) OR (Pollutions, Oil) OR (Oil Spills) OR (Oil Pollutions) OR (Oil Spills Effects) in Title Abstract Keyword AND (Surveys and Questionnaires) OR (Questionnaire Designs) OR (Baseline Surveys) OR (Methods, Survey) AND (Questionnaires) in Title Abstract Keyword - (Word variations have been searched)
Virtual Library of Health (VHL)	(petroleum pollution) OR (oil spill) OR (oil pollution) OR (oil spills effects) AND (surveys and questionnaires) OR (questionnaires) OR (questionnaire designs ) OR (baseline surveys) OR (methods, survey)
Web of Science	(((AB=(oil spills)) OR AB=(oil spills effects)) OR AB=(petroleum pollution)) OR AB=(oil pollution) AND ((((AB=(surveys and questionnaires)) OR AB=(questionnaires)) OR AB=(questionnaire designs )) OR AB=(baseline surveys)) OR AB=(methods, survey)
Embase	(‘petroleum pollution’/exp OR ‘petroleum pollution’ OR ((‘petroleum’/exp OR petroleum) AND (‘pollution’/exp OR pollution)) OR ‘oil spill’:ti,ab,kw OR ‘oil spill effects’:ti,ab,kw OR ‘oil pollution’:ti,ab,kw) AND ([controlled clinical trial]/lim OR [randomized controlled trial]/lim) AND [2011-2023]/py AND ((‘surveys’/exp OR surveys) AND (‘questionnaires’/exp OR questionnaires) OR questionnaire:ti,ab,kw OR ‘questionnaire designs’:ti,ab,kw OR ‘methods, survey’:ti,ab,kw) AND ([controlled clinical trial]/lim OR [randomized controlled trial]/lim) AND [2011-2023]/py
SciELO	(ti:(petroleum pollution)) OR (ti:(oil spill)) OR (ti:(oil spills effects)) OR (ti:(oil pollution)) AND (ti:(surveys and questionnaires)) OR (ti:(surveys)) OR (ti:(questionnaires)) OR (ti:(questionnaire design)) OR (ti:(baseline survey)) OR (ti:(nonrespondent)) OR (ti:(randomized response technique)) OR (ti:(survey methods))
Scopus	(petroleum pollution) OR (oil spill) OR (oil pollution) OR (oil spills effects) AND (surveys and questionnaires) OR (questionnaire) OR (questionnaire designs ) OR (baseline surveys) OR (methods, survey) OR (nonrespondent)

Source: prepared by the authors.

**Figure 1:**
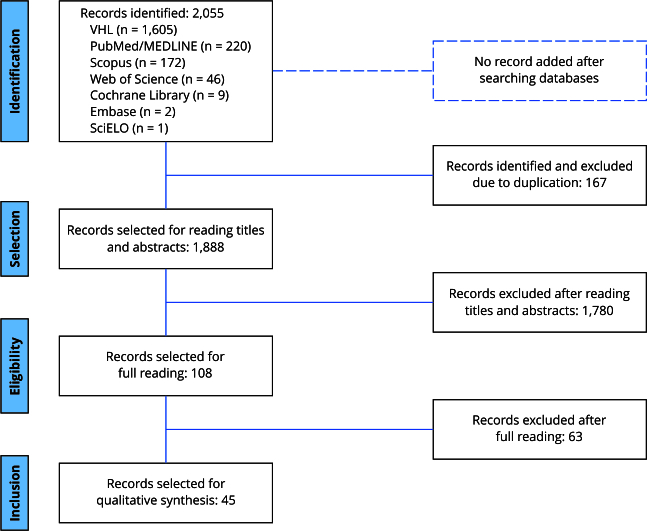
Flowchart indicating the study selection process adapted from *Preferred Reporting Items for Systematic Reviews and Meta-Analyses extension for Scoping Reviews* (PRISMA-ScR).

Source: prepared by the authors.

We identified 2,055 records in the databases. After eliminating 167 duplicates, 1,888 records remained for reading of titles and abstracts. Of these, 1,780 records were excluded for lack of relevant information, resulting in 108 records for full reading. Finally, 45 records were selected for review and narrative synthesis (Figure 1).

We included all original studies in English, Portuguese and Spanish published and available in full, including different methodologies, which should answer the guiding question so as to address the correlation between exposure to oil spill disasters and the consequent impacts, using instruments to measure the effects.

The selected studies, published between 2011 and 2023, reflect the significant increase in research on oil spills after the Deepwater Horizon explosion in 2010, the second largest oil disaster in history and the largest in the aquatic environment ^31^.

We excluded studies of secondary data, reviews, single cases or case series, personal accounts, expert opinions, editorials and similar items. We also excluded academic works (theses, dissertations), books, book chapters, event proceedings, reports and documents. In addition, duplicate or irrelevant studies were removed according to the reading of titles, abstracts or full texts.

For the study selection stage, two independent reviewers were assisted by the Rayyan QCR platform (https://www.rayyan.ai/) in the following steps: reading of titles and abstracts; and reading of full text. In case of disagreement, a third independent reviewer would be required for final decision. In the next step, the extracted information was exported to the Microsoft Excel software (https://products.office.com/), following the characterization of studies selected for sample extraction (author(s); journal; place of publication; objectives; type of study; population; research axis; main results; preliminary training research; level of evidence) and the characterization of instruments used in the studies (identification; instrument application by study; dimensions analyzed; additional information and response patterns as to the instruments used).

The level of evidence used to characterize the studies also followed the recommendations of the Joanna Briggs Institute in five levels and their subclassifications ^32^, with a scale from the level of highest recommendation (1) for experimental studies to the level of lowest recommendation (5) for expert opinions and bench research, the latter not being applicable to the present study, according to the exclusion criteria adopted.

The extracted data were presented in charts, analyzed using descriptive statistics (absolute and relative frequency), followed by narrative synthesis for building the results of this study. Finally, as this study was produced with secondary data, with free access, without direct exposure of humans and/or animals for data collection, it required no submission to the competent Research Ethics Committee.

This study has financial support from the Research Program for SUS: Shared Health Management - PPSUS/Pernambuco (Brazilian National Research Council, Department of Science and Technology of the Secretariat of Science, Technology and Innovation of the Brazilian Ministry of Health, Secretariat of Health of Pernambuco State, Pernambuco Foundation for the Support of Science and Technology [CNPq/Decit/SCTIE/MS/SES/Facepe, acronyms in Portuguese]), the Inova Fiocruz Program - *Strategic Orders: Sustainable and Healthy Territories in the Context of the COVID-19 Pandemic*, and the call for projects and territorialized strategic actions for implementation of the 2030 Agenda, the Presidency of the Oswaldo Cruz Foundation (FIOCRUZ, acronym in Portuguese), and the Brazilian Coordination for the Improvement of Higher Education Personnel (CAPES, acronym in Portuguese; Financial Code 001).

## Results

Regarding the characterization of the selected records (Box 2), it was observed that most studies were carried out in the United States (71.11%), with less frequency in South Korea (13.33%), Nigeria (6.66%), Spain (4.44%), United Kingdom (2.22%) and Brazil (2.22%). The highest volume of publications was in 2019 (20%), followed by 2022 (15.55%) and 2012 (11.11%). Only one study was published in 2023, by researchers from the United States.

**Box 2:** Characterization of the selected studies.

STUDY (YEAR)	LOCATION	OBJECTIVES	STUDY TYPE POPULATION LEVEL OF EVIDENCE	AXIS	MAIN RESULTS	TRAINING RESEARCH
Denic-Roberts et al. ^71^ (2023)	United States	Investigate the risks of long-term neurological conditions among U.S. Coast Guard responders to the 2010 Deepwater Horizon oil spill	Cohort with control group 5,964 respondents and 39,260 non-respondents 3.c	Health	There was a moderate to high increase in the risks of developing long-term neurological conditions, including headaches, migraine, tinnitus and inflammatory nerve conditions	NI
Denic-Roberts et al. ^72^ (2022)	United States	Investigate the symptoms and prevalent cardiovascular conditions and incidents in the Coast Guard cohort exposed to the Deepwater Horizon oil spill	Cohort with control group 5,964 respondents and 39,260 non-respondents 3.c	Health	Higher exposure levels were associated with a higher prevalence of chest pain, in addition to a high risk of mitral valve disorders, palpitations and arterial hypertension	NI
Goldman et al. ^73^ (2022)	United States	Assess the association between exposure to the Deepwater Horizon oil spill, coping/emotional support capacity, and the severity of anxiety symptoms	Cross-sectional 38,361 individuals 4.b	Health	Direct contact with the oil was associated with a higher incidence of increased anxiety symptoms	NI
Ferreira et al. ^74^ (2022)	Brazil	Describe and estimate the impacts of the oil spill on social, economic and health variables of the main affected populations on the Northeast coast	Observational without control group 19,300 individuals in fishing cities; 40,056 individuals in cities 3.e	Health; socioeconomic	Heterogeneous impacts were observed among the socioeconomic indicators of the segments and municipalities assessed, with tourism and fishing regions being the most affected in terms of income reduction and product sales. In addition, health indicators point to symptoms of exogenous intoxication as the main damage	NI
Kwok et al. ^75^ (2022)	United States	Determine if participation in clean-up activities after the Deepwater Horizon disaster was associated with an increased risk of developing hypertension	Cohort with control group 6,846 respondents and 1,505 non-respondents 3.c	Health	It was observed that higher exposure levels were associated with a higher risk of developing hypertension	NI
Lawrence et al. ^76^ (2022)	United States	Assess the primary risks of inhaling oil clean-up chemicals experienced by workers from containment responses following the Deepwater Horizon disaster	Cohort with control group 19,018 respondents and 5,585 non-respondents 3.c	Health	Exposure to chemicals was associated with increased risk of asthma	NI
Oghenetega et al. ^77^ (2022)	Nigeria	Determine the effect of maternal exposure to oil pollution on maternal outcomes in the Niger Delta region of Nigeria	Cohort with control group 1,720 pregnant women between areas of high and low exposure 3.c	Health	It has been observed that women in areas with high exposure to oil pollution have a higher risk of postpartum hemorrhage and premature membrane rupture compared to women in areas of low exposure	NI
Rusiecki et al. ^78^ (2022)	United States	Assess incident respiratory conditions associated with the response to the Deepwater Horizon oil spill	Cohort with control group 5,964 respondents and 39,260 non-respondents 3.c	Health	Higher exposures were associated with higher incidence of sinusitis, chronic obstructive pulmonary disease, dyspnea, and related conditions	NI
Choi et al. ^15^ (2021)	South Korea	Assess the prevalence and risk of psychological symptoms over nine years since the Hebei Spirit oil spill	Cohort with control group 2,013 individuals in high exposure area and 6,495 in low exposure area 3.c	Health	There was a significant association between longer clean-up work time in individuals with lower family income and low education and the risk for depression, anxiety and PTSD	NI
Eleke et al. ^79^ (2021)	Nigeria	Examine the effect of environmental pollution by crude oil on newborn birth outcomes in Rivers State, Nigeria	Cohort with control group 169 records in an affected area and 169 records in an unaffected area 3.c	Health	Increased risks of preterm birth, slower growth, and neonatal morbidity within 6 weeks of birth in environments most affected by oil	NI
Harville et al. ^80^ (2021)	United States	Examine the relation between oil spill exposure and birth outcomes near the Gulf of Mexico	Observational without control group 1,375 women, 503 of whom gave birth to children before and after the spill 3.e	Health	Associations between most indicators of oil spill exposure and pregnancy outcomes were null, despite high levels of contact with oil	NI
Bergstrand & Mayer ^81^ (2020)	United States	Investigate long-term perceptions of community recovery after oil spill	Cohort with control group 351 individuals in areas with high and low exposure to oil spill 3.c	Environmental; health; socioeconomic	The influence of the community on the perception of recovery after the disaster was observed, although reports indicate the perception of little economic and environmental recovery	NI
Bebeteidoh et al. ^82^ (2020)	United Kingdom	Determine the impact of the activities of local crude oil refineries in the Niger Delta on their host communities	Cross-sectional 487 individuals 4.b	Environmental	Impacts on fishing routes and cultivation areas were observed, affecting the livelihoods of fishing and agricultural workers	NI
Oghenetega et al. ^83^ (2020)	Nigeria	Determine the association between oil pollution and miscarriage, stillbirth and infant death in the Niger Delta region	Cohort with control group 782 women in high exposure area and 782 women in low exposure area 3.c	Health	Higher incidence of infant death in the region with high exposure to oil pollution, with no association between high exposure and spontaneous abortion and stillbirth	NI
Parker et al. ^84^ (2020)	United States	Examine the nature and predictors of concern about the continuous impacts of the 2010 Deepwater Horizon oil spill	Observational without control group 903 respondents from a sample of 2,520 individuals 3.e	Health; socioeconomic	Higher exposure to the oil spill was associated with higher levels of concern about the impacts, especially those related to health	NI
Erickson et al. ^85^ (2019)	United States	Assess the relation between exposure to environmental heat and related symptoms among disaster responders of the Deepwater Horizon oil spill	Cross-sectional 3,648 individuals 4.b	Health	Higher heat exposures during oil spill response actions were associated with higher prevalence of heat-related symptoms compared to those with lower exposure	NI
Kaufman et al. ^86^ (2019)	United States	Evaluate the association between direct contact with oil and the severity of depression among Gulf Coast residents following the Deepwater Horizon oil spill and assess the potential moderation of this association by participation of clean-up, self-mastery, or emotional support	Cross-sectional 38,361 individuals 4.b	Health	It was observed that contact with oil was associated with increased severity of depression, especially for those with less self-mastery or emotional support	NI
Krishnamurthy et al. ^87^ (2019)	United States	Assess the association between crude oil exposures and acute neurological symptoms reported by responders to the Deepwater Horizon oil spill	Cohort with control group 4,855 respondents and 44,823 non-respondents 3.c	Health	Exposure to petroleum in isolation or combined with chemicals was moderately associated with increased prevalence of acute neurological symptoms	NI
Nugent et al. ^88^ (2019)	United States	Describe the PTSD profiles among women and the association with the level of exposure to the Deepwater Horizon oil spill	Observational without control group 1,997 women from an initial sample of 2,852 3.e	Health	The study observed 5 profiles of women with PTSD, ranging from milder to more severe levels of symptoms, associated with the degree of exposure	NI
Quist et al. ^19^ (2019)	United States	Examine the association of THC concentrations and containment work classes in the Deepwater Horizon oil spill with the neurobehavioral function among workers	Observational without control group 3,291 respondents from a total sample of 32,608 individuals 3.e	Health	More exposed workers showed greater changes in attention, memory and executive function	NI
Rung et al. ^89^ (2019)	United States	Describe changes in mental health among women after the oil spill and examine their relation to exposure over time	Observational without control group 2,038 women from an initial sample of 2,852 3.e	Health	It was observed that depressive symptoms increased after the oil spill and that the association between economic and physical exposure persisted up to 6 years after the disaster	NI
Strelitz et al. ^90^ (2019)	United States	Assess the associations between the duration of the oil spill clean-up work, residential proximity to the oil spill, and the incidence of self-reported myocardial infarction or fatal coronary heart disease	Observational without control group 21,256 individuals from an initial sample of 32,608 3.e	Health	Residing near the oil spill (vs. residing far away) was associated with heart disease, and longer working hours were associated with increased risk, persisting for 5 years	NI
Strelitz et al. ^91^ (2019)	United States	Assess the relation between exposure to THC used during Deepwater Horizon oil spill response and cleanup and the risk of acute myocardial infarction	Observational without control group 16,814 individuals from an initial sample of 24,375 3.e	Health	Higher levels of exposure to the chemical were associated with a higher risk of acute myocardial infarction, with records of 312 incidents	NI
Werder et al. ^92^ (2019)	United States	Assess associations between blood BTEX levels and symptoms in Gulf Coast residents	Observational without control group 690 individuals from an initial sample of 1,055 3.e	Health	It was observed that half of the subjects had at least one neurological symptom after exposure to the chemical	NI
Alexander et al. ^93^ (2018)	United States	Examine the association between specific exposures observed during oil spill clean-up and acute respiratory symptoms	Cross-sectional 4,855 first responders involved in spill containment efforts 4.b	Health	Higher prevalence of cough, followed by shortness of breath and “wheezing”, suggesting a correlation between exposure and effect	NI
Harville et al. ^94^ (2018)	United States	Examine the association between self-reported exposure to the oil spill and self-reported miscarriage or infertility	Observational without control group 1,620 women, including 443 who were pregnant during the interview 3.e	Health	There was an increased risk of miscarriage for any level of oil exposure and fertility problems in the women most exposed to the spill	NI
Rusiecki et al. ^95^ (2018)	United States	Investigate the potential acute and long-term effects on health resulting from exposure of response workers to oil spills	Cohort with control group 8,696 respondents and 44,823 non-respondents 3.c	Health	Exposure to crude oil has been associated with symptoms related to cough, shortness of breath, itching, headaches, dizziness, diarrhea, stomach pain, nausea/vomiting, burning when urinating, and asthma	NI
Strelitz et al. ^96^ (2018)	United States	Evaluate the relation of clean-up work and proximity to oil spill with self-reported risk of myocardial infarction	Observational without control group 31,109 individuals with no prior history of myocardial infarction 3.e	Health	There were 192 heart attacks during the study period; 151 among the workers. Clean-up work and proximity were suggestively associated with a possible increased risk of non-fatal myocardial infarction	NI
Croisant et al. ^17^ (2017)	United States	Understand physical and mental health effects attributable to the Macondo oil spill	Cross-sectional 324 individuals 4.b	Health	There were changes in self-reported physical and mental health status after the oil spill, disparities in access to healthcare, and associations between mental health and emotional conditions related to movement/unemployment	NI
Harville et al. ^18^ (2017)	United States	Examine the association between self-reported exposure to the physical, social and economic effects of the Gulf oil spill and pregnancy complications	Observational without control group 1,650 women, including 460 pregnant women during the interview 3.e	Health; socioeconomic	No association was observed between exposure to oil spill and hypertensive disorders; however, there was a greater propensity to gestational diabetes	NI
Kwok et al. ^97^ (2017)	United States	Analyze the effects of the Deepwater Horizon disaster on the mental health of individuals involved in oil spill response and clean-up	Cohort with control group 8,968 respondents and 2,225 non-respondents 3.c	Health	Increased prevalence of depression was observed in those individuals involved in the clean-up work	NI
McGowan et al. ^98^ (2017)	United States	Examine associations between potential exposure to dispersants and adverse respiratory, dermal, and eye irritation symptoms	Observational without control group Between 27,659 and 29,468 participants who were interviewed 3.e	Health	There was a significant association between potential exposure to any dispersant and all health outcomes, especially burning nose, throat or lungs, tight chest, and burning eyes	NI
Zilversmit et al. ^99^ (2017)	United States	Compare seafood with the blood levels of Hg and n-3 PUFAs between pregnant and non-pregnant women	Observational without control group 634 women of 1,788 recruited 3.e	Health	Higher levels of Hg were observed in the blood and seafood of pregnant women, in addition to changes in eating behavior, which showed a reduced overall consumption of fish	NI
Nriagu et al. ^100^ (2016)	United States	Determine the prevalence and correlates of measures of health and emotional distress in an area of the Niger Delta, explore the local population’s perception of environmental risks and their influence on emotional distress, and establish relations between exposure to oil pollution and measures of health outcomes	Cross-sectional 600 individuals 4.b	Environmental; health	A high level of suffering was observed in the entire study population. Risk perception was largely by feared hazards, visual cues, and chemosensory cues. Exposure metrics were considered significant predictors of health effects and influencing factors (emotional reactions)	NI
Peres et al. ^101^ (2016)	United States	Characterize individual exposure to the Deepwater Horizon oil spill and examine its association with physical health	Observational without control group 2,852 women from an initial sample of 42,649 3.e	Health	There was a significant relation between high physical-environmental exposure and all physical health symptoms, with stronger associations for burning nose, throat or lungs, sore throat, dizziness and wheezing	NI
Rung et al. ^102^ (2016)	United States	Describe the relation between exposure to oil spill and mental health among women living in the coastal region of southern Louisiana	Observational without control group 2,842 women 3.e	Health	It was observed that economic and physical exposures were significantly associated with depressive symptoms and conflict, while only physical exposure was associated with mental distress	NI
Simon-Friedt et al. ^103^ (2016)	United States	Determine perceived risks within communities exposed to the Deepwater Horizon oil spill	Observational without control group 192 women 3.e	Environmental	There was a significant reduction in seafood consumption, associated with negative environmental perceptions that remain over time	NI
Ha et al. ^104^ (2013)	South Korea	Examine the mental health of children in the area affected by the Hebei Spirit oil spill accident	Cross-sectional 1,362 school-aged children in the region 4.b	Health	There was a significant relation between greater proximity to the affected region and the risk of depression symptoms, with no association for anxiety	NI
Jung et al. ^105^ (2013)	South Korea	Evaluate the respiratory effect of exposure to oil spill on children in Costa Amarela	Observational without control group 436 children from an initial sample of 662 3.e	Health	Children living near the oil spill area showed reduced lung function, increased prevalence of asthma, and hyperresponsiveness	NI
Buttke et al. ^106^ (2012)	United States	Determine the general and mental health needs of the community one year after the Deepwater Horizon oil spill	Cross-sectional Household sampling 4.b	Health	Respondents who reported decreased income after the oil spill were more likely to report mental health symptoms	NI
Gwack et al. ^107^ (2012)	South Korea	Investigate the acute health effects and their related factors among military personnel who participated in the clean-up of the Hebei Spirit oil spill	Cohort with control group 2,050 respondents and 574 non-respondents 3.c	Health	Work in highly-contaminated areas and improper use of personal protective equipment were associated with 17 acute symptoms assessed	NI
Ha et al. ^108^ (2012)	South Korea	Examine the state of exposure and the acute health effects in volunteers in the oil spill clean-up	Observational without control group 565 respondents from an initial sample of 724 3.e	Health	Physical symptoms were associated with longer work durations and significantly higher levels of metabolites after clean-up	NI
Pérez-Pereira et al. ^109^ (2012)	Spain	Study the effect of the Prestige oil spill on the academic performance and classroom behavior of children	Cross-sectional 430 individuals 4.b	Health	The study indicates that, one year after the oil spill, the Prestige disaster had almost no consequences on the aspects evaluated	NI
Zock et al. ^110^ (2012)	Spain	Assess the persistence of respiratory symptoms 5 years after clean-up work in the Prestige oil spill	Cohort with control group 501 exposed fishers and 177 non-exposed fishers 3.c	Health	It was observed that participation in oil clean-up activities may result in respiratory symptoms that persist for up to 5 years after exposure	NI
Cheong et al. ^111^ (2011)	South Korea	Examine the relation between exposure to crude oil and physical symptoms among residents who participated in the oil spill clean-up work	Observational without control group 288 individuals 3.e	Health	Exposure during clean-up work showed associations with physical symptoms, although with no abnormalities in exposure biomarkers in urine	NI

BTEX: benzene, toluene, ethylbenzene, and xylene; NI: not informed; PTSD: post-traumatic stress disorder; PUFA: polyunsaturated fatty acids; THC: total hydrocarbon.

Source: prepared by the authors.

* Levels of evidence: 3.c - cohort study with control group; 3.e - observational study without control group; 4.b - cross-sectional study.

The studies identified were: Observational Studies without Control Group (44.44%; level of evidence 3.e), Cohort Studies with Control Group (33.33%; level of evidence 3.c) and Cross-Sectional Studies (22.22%; level of evidence 4.b).

As for the relation between research objectives and axes of the selected articles, it was observed that most studies focused on the health field (86.66%), with little emphasis on the environment (4.44%) and no exclusive research on socioeconomic aspects. The review found mixed approaches: health and socioeconomic aspects (6.66%); health and the environment (2.22%); and health, the environment and socioeconomic aspects (2.22%). No study mentioned the preliminary training research in the methodological design.

As for the characterization of instruments used by the selected articles (Box 3), it was observed that most studies used their own questionnaire applied through interviews (64.44%), in contrast to the isolated use of other assessment instruments (6.66%). In addition, we identified records that combined different instruments (28.88%).

**Box 3:** Instrument characterization.

STUDY (YEAR)	MEASUREMENT, EVALUATION AND MONITORING INSTRUMENT			
	IDENTIFICATION	INSTRUMENT APPLICATION BY THE STUDY	DIMENSION ANALYZED	ADDITIONAL INFORMATION AND RESPONSE PATTERNS OF THE INSTRUMENTS
Denic-Roberts et al. ^71^ (2023)	Own questionnaire for application in interview method	The instrument considered the exposure to oil spill to establish correlation with a list of self-reported chronic neurological conditions based on the ICD, including migraine, memory and cognition, peripheral, sensitivity, balance and gait disorders	Health Neurological aspects	Binary Scale (always/never): routes of exposure - inhalation; direct contact; ingestion; immersion
				5-Point Scale (never/rarely/sometimes/most of the time/all of the time): routes of exposure - inhalation; direct contact; ingestion; immersion
Denic-Roberts et al. ^72^ (2022)	Own questionnaire for application in interview method	The instrument considered exposure to oil spill to establish correlation with a list of self-reported cardiovascular conditions, based on the ICD, including hypertension, coronary atherosclerosis, ischemic diseases, conduction diseases, dysrithymias, embolism and thrombosis, and other symptoms involving the cardiovascular system	Health Cardiovascular aspects	Binary Scale (always/never): routes of exposure - inhalation; direct contact; ingestion; immersion
				5-Point Scale (never/rarely/sometimes/most of the time/all of the time): routes of exposure - inhalation; direct contact; ingestion; immersion
				3-Point Scale (never/sometimes/most of the time): acute cardiovascular symptoms - chest pain; sudden change in pulse
Goldman et al. ^73^ (2022)	GSPS	Instrument used to collect data regarding the experiences related to the Deepwater Horizon oil spill and the mental health of the community	General Perceptions of exposure and impacts	Binary Scale (yes/no): contact with spilled oil, in 16 items related to this exposure measure
	GAD-7	Instrument used for self-assessment in moderate to severe cases of generalized anxiety. The scores, 0 to 21, were correlated to the data obtained by GSPS	Health Mental and emotional aspects	4-Point Scale (no time (0)/several days (1)/more than half of the days (2)/almost every day (3)): seven questions related to the frequency of anxiety-related problems
Ferreira et al. ^74^ (2022)	Own questionnaire for application in interview method	The authors report the use of four types of questionnaires to generate a specific database, but not found in the search source made available	Multidimensional Socioeconomic and general health aspects	In the methodological details of this study, there is not enough information to characterize elements of the instrument and points evaluated
Kwok et al. ^75^ (2022)	Own questionnaire for application in interview method	The instrument considered exposure to oil spill to establish correlation with blood pressure measures collected during meetings with the respondents	Health Cardiovascular aspects	In the methodological details of this study, there is not enough information to characterize elements of the instrument and points evaluated
Lawrence et al. ^76^ (2022)	Own questionnaire for application in interview method	The instrument considered exposure to oil spill to establish a correlation with the occurrence of respiratory symptoms related to asthma, emphysema and bronchitis. For this study, associated exposure estimations were also performed with dosimetric samples of combustion and air quality	Health Respiratory aspects	5-Point Scale (never/rarely/sometimes/most of the time/all of the time): occurrence of wheezing as suggestive of asthma
				Binary Scale (yes/no): for incident cases of asthma, emphysema and bronchitis diagnosed by medical evaluation
				Binary Scale (yes/no): for exposure to oil spill containment work
Oghenetega et al. ^77^ (2022)	Own questionnaire for application in interview method	The instrument considered exposure to oil spill to establish a correlation with maternal and neonatal outcomes	Health Obstetric and neonatal aspects	Combined Scale (yes/no); (uncontaminated/slightly contaminated/contaminated); (safe/slightly contaminated/unsafe and contaminated/ very unsafe/highly contaminated): for oil pollution exposure characteristics
				Combined Scale (yes/no); (never/daily/once or twice a week/once a week or more/no response/other) & Free Response Patterns: for maternal characteristics and lifestyles - number of pregnancies; previous stillbirth; previous infant death; smoking; alcohol consumption
Rusiecki et al. ^78^ (2022)	Own questionnaire for application in interview method	The instrument considered exposure to oil spill to establish correlation with a list of self-reported respiratory conditions, based on the ICD, including chronic obstructive pulmonary disease, rhinitis, sinusitis, bronchitis, asthma, dyspnea, wheezing, cough, and thoracic symptoms	Health Respiratory aspects	Binary Scale (always/never): routes of exposure - inhalation; direct contact; ingestion; immersion
				5-Point Scale (never/rarely/sometimes/most of the time/all of the time): routes of exposure - inhalation; direct contact; ingestion; immersion
Choi et al. ^15^ (2021)	Own questionnaire for application in interview method	The instrument considered exposure to oil spill through clean-up work and its duration self-reported during the interview to correlate with psychological symptoms	General Perceptions of exposure and impacts	Combined Scale (yes/no/unknown); [Q1 (2 < 12 days), Q2 (12 < 48 days), Q3 (48 < 97 days) and Q4 (97-400 days)]: for responses related to work and duration, respectively
	PDS	The scale used consists of 17 items related to symptoms of PTSD, translated into Korean and also validated	Health Mental and emotional aspects	4-Point Scale (never (0)/a little (1)/very (2)/always (3)): score above 15 was considered risk for PTSD
	CES-D	The scale used consists of 20 items related to depressive symptoms, translated into Korean and also validated	Health Mental and emotional aspects	4-Point Scale (rarely or never (0)/1 or 2 days (1)/3 or 4 days (2)/always (3)): a score above 21 was considered as risk for depression
	PWI-SF	The form used consists of 18 items with questions related to psychosocial well-being, translated into Korean and also validated	Health Mental and emotional aspects	4-Point Scale (never (0)/sometimes (1)/frequently (2)/always (3)): a score above 27 was considered as risk for psychosocial ill-being
	STAI	The inventory used consists of 20 items for evaluation of subjective components related to anxiety, translated into Korean and also validated	Health Mental and emotional aspects	4-Point Scale (never (1)/sometimes (2)/frequently (3)/always (4)): a score above 52 was considered as risk for anxiety
Eleke et al. ^79^ (2021)	Own questionnaire for application in interview method	The instrument considered the effects of exposure to oil spill on gestational, perinatal and neonatal conditions based on the responses collected	Health Obstetric and neonatal aspects	In the methodological details of this study, there is not enough information to characterize elements of the instrument and points evaluated
Harville et al. ^80^ (2021)	Own questionnaire for application in interview method	The instrument considered exposure to oil spill through clean-up work and its duration self-reported during the interview to correlate with the socioeconomic impacts and the impacts on the gestational health of women	Health Obstetric and neonatal aspects	Free Response Patterns: for clean-up work involvement and oil contact, direct exposure, and exposure-related socioeconomic effects
				Free Response Patterns: for outcomes related to the reproductive history and the final outcome of each pregnancy, in addition to birth weight
Bergstrand & Mayer ^81^ (2020)	CERA (*Gulf Coast Module*) adapted	The adaptation of this instrument was used in the approach related to the environment, environmental concern, community optimism and employment variables, in addition to the general effects of the oil spill and the perception of recovery over time	Multidimensional Perception of exposure and environmental impact; socioeconomic and general health aspects	6-Point Scale (completely disagree (0)/agree a little (1)/somewhat agree (2)/moderately agree (3)/ strongly agree (4)/completely agree (5)): for evaluation of community support
				6-Point Scale (none (0)/a little (1)/somewhat (2)/ moderately (3)/very (4)/completely (5)): for perceptions of recovery in four dimensions - economy, environment, community and health
				Free Response Patterns: for subjective interpretations of social cohesion
				Binary Scale (large effects (1)/little or no effect or unaware (0)): to assess perceptions of environmental problems
				Binary Scale (better place/worse place): to evaluate optimism in relation to the community
				Binary Scale (yes/no): for information related to race, marital status, family income, and education level
Bebeteidoh et al. ^82^ (2020)	Own questionnaire for application in interview method	The instrument considered the impact of the activities of local crude oil refineries, on the community and the environment, perceived by individuals	Environmental Perceptions of risks and impacts	6-Point Scale (completely disagree (0)/agree a little (1)/somewhat agree (2)/moderately agree (3)/strongly agree (4)/completely agree (5)): for environmental impact assessment in 6 dimensions - farmlands, farm yields, fishing, fishing yields, water pollution, and waste
Oghenetega et al. ^83^ (2020)	Own questionnaire for application in interview method	The instrument considered sociodemographic and socioeconomic characteristics, in addition to characteristics related to exposure to oil pollution and obstetric history of pregnant women	Health Obstetric and neonatal aspects	In the methodological details of this study, there is not enough information to characterize elements of the instrument and points evaluated
Parker et al. ^84^ (2020)	Own questionnaire for application in interview method	The instrument considered levels of concern and traumatic experiences of individuals exposed to continuous impacts since the oil spill	Multidimensional Socioeconomic and general health aspects	4-Point Scale (not at all concerned (0)/somewhat concerned (1)/moderately concerned (2)/very concerned (3)): for persistent concern in relation to continuous impacts
				Free Response Patterns: for information about the mobility of individuals between places exposed to the spill
				Free Response Patterns: for exposure-related memories
	*Trauma History Screen* (adapted)	The adaptation of this instrument was used in the approach related to victimization events that occurred in adulthood, distributed in 12 items to count the traumatic experiences of adulthood	Health Mental and emotional aspects	Binary Scale (since the age of 18/in the last year): for evaluation of traumatic experiences over time
Erickson et al. ^85^ (2019)	Own questionnaire for application in interview method	The instrument considered exposure to oil spill to establish correlation with heat-associated physical symptoms	Health General aspects	5-Point Scale (never/rarely/sometimes/most of the time/all of the time): routes of exposure - inhalation; direct contact; ingestion; immersion
				Binary Scale (always/never): for the use of personal protective equipment
				Free Response Patterns: for information about heat-related symptoms
Kaufman et al. ^86^ (2019)	Own questionnaire for application in interview method	The instrument considered exposure to oil spill to establish correlation with depression symptoms, as well as factors related to emotional support, burdens and participation in oil spill clean-up efforts	Health Mental and emotional aspects	Binary Scale (yes/no): for contact with spilled oil and participation in clean-up efforts
				5-Point Scale (never/rarely/sometimes/most times/all the time): for assessment of emotional support in 5 contexts during efforts
	PHQ-8	The validated instrument was used for assessment of self-reported frequency of depression symptoms in respondents	Health Mental and emotional aspects	4-Point Scale (no time (0)/several days (1)/more than half of the days (2)/almost every day (3)): a score above 10 was considered as risk for depression
Krishnamurthy et al. ^87^ (2019)	Own questionnaire for application in interview method	The instrument considered exposure to oil spill and chemicals related to clean-up efforts to establish correlation with acute neurological symptoms	Health Neurological aspects	5-Point Scale (never/rarely/sometimes/most of the time/all of the time): for the frequency of exposure to crude oil/oily water, in addition to oil dispersants
				3-Point Scale (never/sometimes/most of the time): for self-reported neurological symptoms
				Free Response Patterns: for sociodemographic data and oil and dispersant exposure data
Nugent et al. ^88^ (2019)	Own questionnaire for application in interview method	The instrument considered exposure to oil spill to establish correlation with symptoms and occurrence of PTSD	Health Mental and emotional aspects	Free Response Patterns: for sociodemographic data, and data related to exposure and perception of physical health status
				Binary Scale (yes/no): for the ability to smell oil, loss of household income, and impact on financial situation
				5-Point Scale (no influence/a positive influence/an influence/a negative influence/a very negative influence): for specific assessment of financial situation
	*Life Events Checklist*	20-item instrument to assess potentially traumatic experiences throughout life	Health Mental and emotional aspects	5-Point Scale (happened to me/witnessed it/learned about it/not sure/not applicable): for specific assessment of financial situation
Quist et al. ^19^ (2019)	Own questionnaire for application in interview method	The instrument considered exposure to oil spill and chemicals related to clean-up efforts to establish correlation with neurobehavioral symptoms	Health Mental and emotional aspects	In the methodological details of this study, there is not enough information to characterize elements of the instrument and points evaluated
	BARS	The instrument was used for neurobehavioral assessment, including a series of tests with simple verbal instructions and response graphs. Each test has one or more outcome measures to assess factors such as response latency, error, and correct responses	Health Mental and emotional aspects	Continuous Performance Test: to assess sustained visual attention and short-term memory
				Digit Span Test: to assess attention and memory
				Sample Correspondence Test: to assess visual memory
				Digit Symbol Substitution Test: to assess executive function and coding
				Simple Reaction Time Test: to evaluate response speed
				Finger Tapping Test: to assess motor speed and coordination
				Progressive Proportion Test: to assess effort-related motivation
Rung et al. ^89^ (2019)	Own questionnaire for application in interview method	The instrument considered exposure to oil spill to establish correlation with physical and socioeconomic impacts	General Perceptions of exposure and impacts	In the methodological details of this study, there is not enough information to characterize elements of the instrument and points evaluated, although the authors mention the grouping of items in “economic exposure” and “physical exposure” to record the frequency of reports
	CES-D	Instrument designed for epidemiological studies, which allows the evaluation of depressive symptoms and mood variations. In this study, the scale was used as a continuous measure of symptoms	Health Mental and emotional aspects	4-Point Scale (rarely or never (0)/1 or 2 days (1)/3 or 4 days (2)/always (3)): a score above 16 was considered as risk for depression
	K6	Non-specific psychological distress assessment instrument used in the screening of mood and anxiety disorders. In this study, the scale was used as a continuous measure of symptoms	Health Mental and emotional aspects	5-Point Scale (never (1)/rarely (2)/sometimes (3)/most of the time (4)/all of the time (5)): a score above or equal to 13 was considered as probable severe mental distress
Strelitz et al. ^90^ (2019)	Own questionnaire for application in interview method	The instrument considered exposure to oil spill and duration of clean-up work to establish correlation with occurrence of a first event of self-reported or fatal heart disease, in the latter case, evaluated by the National Death Index	Health Cardiovascular aspects	Free Response Patterns: for data on sociodemographic dimensions, duration of exposure, duration of clean-up work, and residential proximity, in addition to diagnosis of a cardiac event
Strelitz et al. ^91^ (2019)	Own questionnaire for application in interview method	The instrument considered exposure to oil spill based on self-reported activities, associated with general hydrocarbon markers, to correlate with the occurrence of cardiac events	Health Cardiovascular aspects	Free Response Patterns: for data related to activities and work patterns involving exposure, as well as outcomes of interest for a first cardiac incident and demographic and lifestyle factors
Werder et al. ^92^ (2019)	Own questionnaire for application in interview method	The instrument considered the occurrence of neurological symptoms of respondents to correlate with exposure to chemicals related to spilled oil clean-up work	Health Neurological aspects	5-Point Scale (never/rarely/sometimes/most of the time/all of the time): for self-reported frequency of neurological symptoms
				Free Response Patterns: for sociodemographic information and information related to exposure to chemicals
Alexander et al. ^93^ (2018)	Own questionnaire for application in interview method	The instrument considered the occurrence of acute respiratory symptoms of respondents to correlate with exposure to oil spill and chemicals related to clean-up work	Health Respiratory aspects	5-Point Scale (never/rarely/sometimes/most of the time/all of the time): for the frequency of exposure to crude oil/oily water and dispersants in four contexts - inhalation, direct skin contact, ingestion, and submersion
				3-Point Scale (never/sometimes/most of the time): for self-reported acute respiratory symptoms
Harville et al. ^94^ (2018)	Own questionnaire for application in interview method	The instrument considered the occurrence of spontaneous abortions and infertility among the women interviewed to correlate with exposure to oil spill	Health Obstetric and neonatal aspects	Combined Scale (some/none); (none/some/very much): for financial/income consequences, direct contact with oil, trauma related to oil spill and loss of shore use
				Binary Scale (yes/no): for the occurrence of behavioral changes related to oil spill
				Free Response Patterns: for information related to the reproductive history of the women interviewed, in addition to their behavioral changes
Rusiecki et al. ^95^ (2018)	Own questionnaire for application in interview method	The instrument considered the occurrence of acute respiratory/neurological/genitourinary/cardiovascular symptoms of the individuals interviewed to correlate with exposure to oil spill and clean-up work-related chemicals	Health General aspects	Binary Scale (always/never): for the self-reported relation between exposure and health effects
				5-Point Scale (never/rarely/sometimes/most of the time/all of the time): for the frequency of exposure to crude oil/oily water and dispersants, in addition to exhaust gases
				3-Point Scale (never/sometimes/most of the time): for self-reported acute respiratory/neurological/genitourinary/cardiovascular symptoms
				Free Response Patterns: for information related to work and exposure to oil spill, in addition to the use of personal protective equipment, experienced acute symptoms and lifestyle factors
Strelitz et al. ^96^ (2018)	Own questionnaire for application in interview method	The instrument considered exposure to oil spill based on self-reported activities, associated with residential proximity, to correlate with the occurrence of non-fatal myocardial infarction	Health Cardiovascular aspects	Free Response Patterns: for data related to activities and work patterns involving exposure, in addition to outcomes of interest for non-fatal myocardial infarction and demographic and lifestyle factors
				Binary Scale (yes/no): for participation in clean-up work, work with burning crude oil and interruption due to thermal exposure
				Binary Scale (direct/indirect): for residential proximity to the location exposed to oil spill
				4-Point Scale (1-30 days/31-90 days/91-180 days/> 180 days): for duration of clean-up work
Croisant et al. ^17^ (2017)	Own questionnaire for application in interview method	The instrument considered the general perception of health and associated social factors to correlate with exposure to oil spill and other measuring instruments	Geral Perceptions of exposure and impacts	5-Point Scale (very poor/poor/regular/good/very good): for items related to the general perception of health
				Binary Scale (yes/no): for the diagnosis of hypertension, diabetes, heart diseases, brain diseases or cancer, in addition to access to healthcare services and economic and life conduct impacts
				5-Point Scale (never/rarely/sometimes/most of the time/all of the time): for items related to social support
	GAD-7	Instrument used for self-assessment in cases of generalized anxiety. Scores for the seven items were summed (range 0 to 21) and interpreted as minimal anxiety (0 to 4), mild anxiety (5 to 9), moderate anxiety (10 to 14), and severe anxiety (15 to 21)	Health Mental and emotional aspects	4-Point Scale (0-1 day (0)/2-6 days (1)/7-11 days (2)/12-14 days (3)): 7 questions related to the frequency of anxiety-related problems
	PHQ-8	Instrument used for self-assessment of the frequency of depression symptoms in respondents. Scores for depressive symptoms were interpreted as none (0-4), mild (5-9), moderate (10-14), moderately severe (15-19), and severe (20-24)	Health Mental and emotional aspects	4-Point Scale (0-1 day (0)/2-6 days (1)/7-11 days (2)/12-14 days (3)): 7 questions related to the frequency of problems related to symptoms of depression
	PC-PTSD	Four-item instrument used for the assessment of PTSD. Presence was interpreted as a score of 3 or more	Health Mental and emotional aspects	Binary Scale (yes/no): for symptoms of the disorder observed during the last 30 days
	Self-Mastery Scale	4-item instrument used to assess resilience and coping capacity. Higher scores indicating higher levels of self-mastery	Health Mental and emotional aspects	5-Point Scale (completely disagree (1)/agree a little (2)/somewhat agree (3)/moderately agree (4)/completely agree (5)): for items related to self-mastery
Harville et al. ^18^ (2017)	Own questionnaire for application in interview method	The instrument considered the occurrence of physical and socioeconomic effects, as well as pregnancy complications, among the women interviewed to correlate with exposure to oil spill	Multidimensional Socioeconomic and obstetric health aspects	Combined Scale (some/none); (none/some/very much): for financial/income consequences, direct contact with oil, trauma related to oil spill and loss of shore use
				Free Response Patterns: for information related to involvement in clean-up work and direct exposure to oil spill
				Free Response Patterns: for information related to the reproductive history of the women interviewed
Kwok et al. ^97^ (2017)	Own questionnaire for application in interview method	The instrument considered the occurrence of mental health effects among respondents to correlate with exposure to oil spill	Health Mental and emotional aspects	Free Response Patterns: for information related to involvement in clean-up work and direct exposure to oil spill
	PHQ-8	Instrument used for self-assessment of the frequency of depression symptoms in respondents. Scores of 10 or higher suggest a likely indication of moderate to severe depression	Health Mental and emotional aspects	4-Point Scale (0-1 day (0)/2-6 days (1)/7-11 days (2)/12-14 days (3)): 7 questions related to the frequency of problems related to symptoms of depression
	PC-PTSD	4-item instrument used for the assessment of PTSD. Presence was interpreted as a score of 3 or more	Health Mental and emotional aspects	Binary Scale (yes/no): for symptoms of the disorder observed during the last 30 days
McGowan et al. ^98^ (2017)	Own questionnaire for application in interview method	The instrument considered the occurrence of respiratory, ocular and dermal symptoms among respondents to correlate with exposure to oil spill	Health General aspects	Binary Scale (always/never): for respiratory symptoms, ocular and dermal irritation, and exposure through work and to dispersants used in clean-up
				5-Point Scale (never/rarely/sometimes/most of the time/all of the time): for the frequency of symptoms presented
Zilversmit et al. ^99^ (2017)	Own questionnaire for application in interview method	The instrument considered the frequency of seafood consumption among respondents to correlate with levels of biomarkers in blood after exposure	Health General aspects	8-Point Scale (never/less than once a month/once a month/2-3 times a month/1-2 times a week/3-4 times a week/5-6 times a week/1 or more times a day): for the frequency of seafood consumption
Nriagu et al. ^100^ (2016)	GHQ adapted	The adaptation of this instrument was used in the approach related to the limitation of functional capacity, distributed in 9 items to assess the level of limitation for typical daily activities, such as buying groceries, fishing and farming. The scale’s total score (ranging from 4 to 36) was classified into 4-9 (low), 10-20 (medium), and 21-36 (high)	Health General aspects	4-Point Scale (less than normal (1)/no more than usual (2)/slightly more than normal (3)/much more than normal (4)): 7 questions related to the frequency of problems related to symptoms of depression
	EEQs	4 environmental exposure questions to assess the environmental exposure dimension in terms of residential distance and frequency of direct contact with oil pollution	Environmental Perceptions of risks and impacts	Q1 and Q2 (< 50m, 50-100m, 100-500m and > 500m): for residential distance to the location of visible pollution and gas burning facilities, respectively; Q3 4-Point Scale (never (1)/1-5 times (2)/5-10 times (3)/more than 10 times (4)): for frequency of exposure to pollution; Q4 4-Point Scale (uncontaminated (1)/somewhat contaminated (2)/very contaminated (3)/highly contaminated (4)): for drinking water pollution level
	Risky events self-report questionnaire	The adaptation of this scale was used in the approach related to perceived environmental risk. The scale’s total score (ranging from 4 to 28) was categorized into 4-10 (low), 11-19 (medium), 20-28 (high)	Environmental Perceptions of risks and impacts	4-Point Scale (not at all concerned (1)/somewhat concerned (2)/moderately concerned (3)/very concerned (4)): 7 questions related to perceived environmental risk
	ERT	Standardized scale for the assessment of environmental risk tolerance, based on 11 statements related to oil pollution. The scale was subcategorized into 0-5 (minimum tolerance), 6-15 (low tolerance), 16-25 (medium tolerance), and 26-44 (high tolerance). Reverse score on the scales gives an indication of environmental risk intolerance	Environmental Perceptions of risks and impacts	4-Point Scale (not at all concerned (1)/somewhat concerned (2)/moderately concerned (3)/very concerned (4)): 7 questions related to perceived environmental risk
	EHA	Scale for assessment of annoyance associated with the perception of environmental hazard, based on 12 general questions of adverse environmental events. The scale’s total score was categorized into 4-10 (minimum), 11-20 (low), 21-30 (medium), and 31-48 (high) for data analysis	Environmental Perceptions of risks and impacts	4-Point Scale (not at all concerned (1)/somewhat concerned (2)/moderately concerned (3)/very concerned (4)): for questions related to aspects of annoyance after perception of environmental hazard
	PSW	Inventory for assessment of intensity and excess worry about a specific content, in this case, oil pollution. The potential total score of 52 was subdivided into 4-10 (minimum), 11-20 (low), 21-35 (medium), and 36-52 (high)	Health Mental and emotional aspects	4-Point Scale (not at all concerned (0)/somewhat concerned (1)/moderately concerned (2)/very concerned (3)): for questions related to the pathological concern associated with oil pollution
	HSI	Scale used to assess the symptomatic burden of diseases in families of participants. Responses were summed for each individual, resulting in a total score ranging from 0 to 44. The score was subdivided into 0-5 (minimum), 6-15 (low), 16-25 (medium), and 26-44 (high)	Health Mental and emotional aspects	Binary Scale (yes/no): for related general symptoms after exposure
Peres et al. ^101^ (2016)	Own questionnaire for application in interview method	The instrument considered the occurrence of general symptoms among respondents to correlate with exposure to oil spill	Health Genreal aspects	Combined Scale (yes/no); (most affected/equal/least affected)/(very negative/somewhat negative/somewhat positive/very positive/no influence); (no strength/somewhat strong/moderately strong/very strong/completely strong); (never/somewhat/sometime/most of the time/always): for questions related to oil spill exposure
				5-Point Scale (never/rarely/sometimes/most of the time/all of the time): for the frequency of symptoms presented
Rung et al. ^102^ (2016)	Own questionnaire for application in interview method	The instrument considered the effects of exposure to oil spill to correlate with mental health outcomes	General Perceptions of exposure and impacts	Binary Scale (yes/no): for economic consequences related to oil spill and its exposure levels
				Binary Scale (negative influence/positive influence): for the influence of oil spill on family financial situation
	CES-D	The scale used consists of 20 items related to depressive symptoms	Health Mental and emotional aspects	4-Point Scale (rarely or never (0)/one or two days (1)/three or four days (2)/always (3)): the cutoff score for depressive symptoms was 16
	K6	Non-specific psychological distress assessment instrument used in the screening of mood and anxiety disorders	Health Mental and emotional aspects	5-Point Scale (never (1)/rarely (2)/sometimes (3)/most of the time (4)/all of the time (5)): a score above or equal to 13 was considered as probable severe mental distress
Simon-Friedt et al. ^103^ (2016)	Own questionnaire for application in interview method	The instrument considered specific risks perceived within communities after exposure to oil spill	Environmental Perceptions of risks and impacts	In the methodological details of this study, there is not enough information to characterize elements of the instrument and points evaluated
Ha et al. ^104^ (2013)	CDI	Korean version of the inventory for measuring depression symptoms in children, consisting of 27 questions scored from 0 to 2	Health Mental and emotional aspects	3-Point Scale (slightly (0)/moderately (1)/very (3)): the cutoff score for depression symptoms being greater than or equal to 22
	SAIC	Korean version of the inventory for measuring children's anxiety symptoms, consisting of 20 questions scored from 1 to 3	Health Mental and emotional aspects	3-Point Scale (sometimes (1)/often (2)/always (3)): the cutoff score for anxiety symptoms being greater than or equal to 41
Jung et al. ^105^ (2013)	ISAAC modified	Korean version of the questionnaire used for evaluation of asthma-related characteristics, which was correlated with other information associated with allergic condition and pulmonary function test	Health Respiratory aspects	Binary Scale (yes/no): for information related to previous diagnosis of asthma and occurrence of wheezing in the last 12 months
Buttke et al. ^106^ (2012)	CASPER	Instrument used to collect household information on the needs of an affected community after a disaster, involving data related to physical and mental health	Health Mental and emotional aspects; general aspects	The questions were adapted from other sources such as the CDC’s BRFSS, the PHQ-2 (depressive symptoms), and the GAD-2 (anxiety symptoms)
Gwack et al. ^107^ (2012)	Own questionnaire for application in interview method	The instrument considered the occurrence of acute neurological, respiratory, dermatological, ophthalmological and general symptoms among respondents to correlate with exposure to oil spill	Health General aspects	In the methodological details of this study, there is not enough information to characterize elements of the instrument and points evaluated.
Ha et al. ^108^ (2012)	Own questionnaire for application in interview method	The instrument considered the occurrence of general physical symptoms among respondents to correlate with exposure to oil spill and levels of urinary metabolites associated with chemicals used in clean-up work	Health Genereal aspects	Combined Scale (1 day/more than 1 day); (directly/indirectly/other); (none/somewhat/very/deep): for questions related to oil spill exposure
				Binary Scale (yes/no): for the various physical symptoms self-reported by respondents
				Free Response Patterns: for information related to sociodemographic issues, lifestyle habits, prior history and use of personal protective equipment
Pérez-Pereira et al. ^109^ (2012)	CBI	Inventory used to assess the behavior of school-aged children and preteens in the classroom	Health Mental and emotional aspects	Composed of 5 subscales: intelligent behavior (verbal intelligence, creativity and curiosity); extroversion/introversion, consideration/hostility, independence/dependence, and concentration/distraction. In the methodological details of this study, there is not enough information to characterize elements of the instrument and points evaluated
	Questionnaire adapted from Palinkas et al. ^112^	Adaptation used to assess the degree of exposure to disaster as a risk index measured by residential proximity to event location	General Perceptions of exposure and impacts	In the methodological details of this study, there is not enough information to characterize elements of the instrument and points evaluated, although the authors mention the validation by a 6-point scale
	FACES-II	Scale used to evaluate data associated with family issues, comprising 16 items on cohesion and 14 items on adaptability	Health Mental and emotional aspects	In the methodological details of this study, there is not enough information to characterize elements of the instrument and points evaluated
	CSCY	Scale used to assess personal protection/vulnerability and coping strategies	Health Mental and emotional aspects	Composed of 4 subscales: assistance seeking; problem solving; evasive cognitive strategies; evasive behavioral strategies. In the methodological details of this study, there is not enough information to characterize elements of the instrument and points evaluated
Zock et al. ^110^ (2012)	Own questionnaire for application in interview method	The instrument considered the persistence of respiratory symptoms among respondents to correlate with exposure to oil spill	Health Respiratory aspects	Binary Scale (yes/no): for respiratory symptoms and use of inhaled and oral medications in the last 12 months
				3-Point Scale (never/previous/current): for smoking
				Free Response Patterns: for information related to involvement in clean-up work and direct exposure to oil spill
Cheong et al. ^111^ (2011)	Own questionnaire for application in interview method	The instrument considered the occurrence of physical symptoms among respondents to correlate with exposure to oil spill and rates of urinary metabolites related to chemicals	Health General aspects	4-Point Scale (never/little/very/deep): for the frequency of subjective physical symptoms, general characteristics and prior history
				4-Point Scale (less than 10 days/10 to less than 13 days/13 to less than 20 days/20 or more days): for the frequency of exposure to crude oil

BARS: *Behavioral Assessment and Research System*; BRFSS: *Behavioral Risk Factor Surveillance System*; CASPER: *Community Assessment for Public Health Emergency Response*; CBI: *Classroom Behavior Inventory*; CDC: Centers for Disease Control and Prevention; CDI: *Children’s Depression Inventory*; CERA: *Community and Environment in Rural America*; CES-D: *20-item Center for Epidemiological Studies Depression Scale*; CSCY: *Coping Scale for Children and Youth*; EEQs: *Environmental Exposure Questions*; EHA: *Environmental Hazard Annoyance*; ERT: *Environmental Risk Tolerance*; FACES: *Family Adaptability and Cohesion Evaluation Scales*; GAD-2: *2-item Generalized Anxiety Disorder*; GAD-7: *7-item Generalized Anxiety Disorder*; GHQ: *General Health Questionnaire*; GSPS: *Gulf States Population Survey*; HSI: *Health Symptoms Inventory*; ICD: International Classification of Diseases; ISAAC: *International Study of Asthma and Allergies in Childhood Questionnaire*; K6: *Kessler-6*; PC-PTSD: *Primary Care PTSD Screen*; PDS: *Posttraumatic Diagnostic Scale*; PHQ-2: *2-item Patient Health Questionnaire*; PHQ-8: *8-item Patient Health Questionnaire*; PSW: *The Penn State Worry Questionnaire*; PTSD: post-traumatic stress disorder; PWI-SF: *Psychosocial Well-Being Index-Short Form*; SAIC: *State-Trait Anxiety Inventory for Children*; STAI: *State-Trait Anxiety Inventory*.

Source: prepared by the authors.

In total, 75 records of use of instruments were observed. In addition to own questionnaires, we identified 29 different instruments to assess the impacts of oil spills. Among them, the *20-item Center for Epidemiological Studies Depression Scale* (CES-D) and the *8-item Patient Health Questionnaire* (PHQ-8) were the most used, each in three studies. They were followed by the *7-item Generalized Anxiety Disorder* (GAD-7), the *Kessler-6* (K6), and the *Primary Care PTSD* (PC-PTSD) screen, each in two studies. Only four instruments focused on environmental aspects and respondents’ perceptions: *Community and Environment in Rural America Gulf Coast Module*, *Environmental Exposure Questions*, *Risky Events Self-Report Questionnaire* and *Environmental Risk Tolerance*. Other instruments are shown in Box 3.

Nine articles provided insufficient information on the instruments used, limiting a detailed analysis. The use of binary scales, Likert-like scales, combined scales and free responses was observed in the design of the instruments (Box 3).

Among the dimensions analyzed by the instruments (Box 3), there was a predominance of focus on mental and emotional aspects in the health dimension (41.33%), followed by general aspects of physical health (13.33%), and cardiovascular, respiratory and obstetric/neonatal aspects (6.66% each). Neurological aspects were addressed in 2.66% of the studies.

In the general dimension, we analyzed perceptions of exposure and impact (8%), while, in the environmental dimension, we evaluated perceptions of environmental risks and impacts (8%). Multidimensional variables were also identified to assess socioeconomic and general health aspects (2.66%), and socioeconomic aspects with obstetric health, perception of exposure and environmental impact (1.33% each).

Considering Boxes 2 and 3 and the objectives of the articles, exposure to petroleum/crude oil and containment responses are associated with the following findings:

(a) In health: increased neurological and behavioral disorders, such as anxiety disorder, depression, and post-traumatic stress disorder (PTSD); cardiovascular and respiratory disorders, such as hypertension, heart attack, asthma, and lung diseases; and adverse obstetric and neonatal events, such as miscarriages and stillbirths.

(b) In the environment: perceptions of increased risk associated with exposure and impacts on marine life, leading to reduced seafood consumption.

(c) In the socioeconomic context: negative impacts on tourism and fishing, loss of livelihood for local workers and changes in cultivation routes, highlighting the importance of social cohesion in recovering damage.

## Discussion

The studies identified show a variety of instruments used in oil spill disasters, although most adopted their own models of research groups and scale-based data collection methods to examine impacts. These impacts affect both the environment and humans, especially the coastal population, and nearby territories, causing major vulnerability ^33^. This highlights the importance of understanding changes in socioeconomic dynamics, in the occupation of spaces and in the determination of the health-disease process in these areas ^34^.

When comparing oil spill-related disaster management with that of other natural, technological and chemical disasters, both potential and limitations are found. As for potential, we note improved coordination and rapid response, the use of advanced technologies, and effective training of teams. Clear communication strategies and collaboration between institutions also optimize resources and promote an integrated response. However, limitations such as inadequate assessment and management can cause delays in access to affected areas, increased numbers of victims, psychological suffering, loss of coordination in relief actions, cultural destruction, greater vulnerability and migration in search of resources ^35^.

Therefore, it is crucial to continuously improve assessment methodologies to address the diversity of scenarios and the complexity of impacts on affected communities. The analysis of instruments used, especially in disasters caused by oil spills, shows the importance of tools such as CES-D, PHQ-8, GAD-7, K6 and PC-PTSD. These instruments are effective for screening and monitoring post-disaster psychological conditions, being standardized and easy to apply to trace symptoms of depression, anxiety and PTSD. However, despite their usefulness, these instruments may not provide a complete diagnosis and may not represent the complexity of the experiences lived by the affected populations, as well as the social, economic and cultural factors that contribute to the development of mental disorders ^36^ ^,^ ^37^ ^,^ ^38^ ^,^ ^39^ ^,^ ^40^ ^,^ ^41^.

In the context of health, exposure to petroleum is associated with physical and mental, genotoxic and endocrine symptoms with different severities in various organic systems. In addition, both acute and chronic intoxication increase mental health vulnerability, especially in individuals affected by the destruction of territories and loss of survival mechanisms ^42^. We note the predominance of articles focused on mental health, justified by the concern with increased disorders in the survivors’ life, work, family and social life, as well as with impacts on subsistence activities and economic loss ^43^.

The instruments also showed perceptions about environmental impacts and risks, in addition to negative effects on socioeconomic conditions of exposed populations, similar to those found in studies on occupation of territories by polluting industries and environmental unsustainability of these activities ^44^.

Importantly, the articles selected for this review do not address the intimate aspects of environmental injustices and socioeconomic vulnerability caused by exposure to oil. This contributes to a biased knowledge production that hides risk contexts and is unfavorable to human groups that are vulnerable in this impact assessment design model ^10^.

The gap in studies on the perceptions and responses of communities affected by oil spills can lead to inadequate interventions and deficient policies, aggravating inequalities and injustices and intensifying the impact of environmental racism. This review shows the need for an interdisciplinary and systemic approach, with integrated public policies, to understand the relation between health, the environment, and social and economic aspects and to address the adverse effects on living conditions and territory occupation.

In addition, it is suggested that the coordination between the different levels of government should be strengthened, especially at the municipal level, where the formulated policy is directly implemented, strengthening social participation and enabling local communities to collaborate in building solutions for their specific situations - in this case, dealing with disasters ^45^ ^,^ ^46^ ^,^ ^47^.

Given the challenges in managing disasters, it is important to adopt measures such as standardization of rapid response protocols, efficient environmental monitoring systems, and effective inspection mechanisms, integrating these tools with decision-making processes in a context of democratic governance ^48^ ^,^ ^49^ ^,^ ^50^.

Discussion on the research axes - addressed here in a conceptual field - enables understanding causalities and determinations of the impacts caused by human activity, in an attempt to establish equitable and sustainable solutions ^51^. Accordingly, we note the highly polluting characteristics of the process of producing oil and its derivatives, as well as the potential damages in several aspects, such as culture, which drive, in isolation, approaches of studies on impacts ^52^.

In dealing with oil spill disasters, which affect physical, social and economic aspects of populations, community recovery requires integrated efforts to understand risks to health and community sustainability. Therefore, the use of instruments that provide systematic and detailed data collection is advocated, for a complete understanding of the impacts and effectiveness of recovery actions.

The investigation of the Deepwater Horizon explosion found that difficulties in containing the disaster were related to inadequate estimates of oil flows and poor assessment of the risks of exposure and seafood consumption. Fishing activities and the seafood industry, which are essential to the local economy, were significantly affected by the spills. In addition, there is a lack of information on training and specialization in the assessment of environmental exposure of the professionals involved ^53^ ^,^ ^54^ ^,^ ^55^.

There was a lack of studies that integrate the assessment and monitoring of events, especially considering social issues beyond health. This gap can be attributed to the biomedical paradigm, which tends to resist approaches that include subjectivity, values and symbolic aspects of social relations, focusing mainly on the application of research instruments ^56^.

It is important to note that, although this study has focused on the subject of oil spills, other events involving chemicals, such as pesticides, are considered in the scope of this discussion, as these also produce significant socioeconomic and environmental impacts, which are of interest to public health. In this sense, we identified dynamics of exposure combined with consequences for the organic systems of individuals and the socioeconomic structures of affected areas, also associated with mechanisms that produce vulnerability and injustices in a constant cycle of degradation ^57^ ^,^ ^58^. Therefore, it is necessary to consider the risks of chemical accidents as a public health issue.

Industrial development and chemical handling have catastrophic potential that drives the need for accident and disaster planning and prevention policies, with emergency control and safety measures for each process ^59^. Moreover, in the context of exposure to toxic substances, toxicological information centers play a crucial role in the management of serious incidents while treatment services reach victims. To promote appropriate policies, it is necessary to adapt this tool to ensure the efficient flow of information, notifications and referrals between diagnostic, laboratory, statistical, epidemiological and community surveillance subsystems ^60^.

The predominance of observational methods (analytical and descriptive) in the selected studies reflects the traditional epidemiological interest in establishing causality between events, especially between contextual factors and health repercussions. This reinforces the biomedical contribution, while ignoring the importance of integrating social aspects into the construction of scientific knowledge ^61^.

Although observational studies are more adequate to assess the incidence of events, at a lower cost, it is important to consider the occurrence of confounding factors between the groups under observation, during the construction of the methodological design, especially for the proper application of instruments ^62^. Moreover, we note their lower degree of evidence and recommendation when compared to experimental or quasi-experimental studies, in addition to the need to analyze the methodological quality by different strategies, considering the particularities existing in observational cohort studies with a control group, without a control group, and cross-sectional as presented here ^32^ ^,^ ^63^.

A relevant point is the researchers’ distance from the objects of study and the affected communities. This distance is evidenced by the lack of preliminary research that guides the methodological design and the choice of assessment instruments. The relation between the method and the object is crucial to define the scientific approach, the appropriate tool and the results, ensuring an integrated and concrete view of the issue ^64^.

In other words, the choice of assessment instruments for a scientific method must be preceded by immersion and approach to the reality experienced, to understand it and enable its transformation; not just its critical and fragmented analysis ^65^. Thus, for a more holistic and accurate understanding, and better analysis of impacts, greater involvement of communities and immersion of researchers in affected territories is suggested.

The preference for questionnaires in the reviewed studies seems to be justified by the search for information directly from respondents, allowing inferences with lower costs, in less time and with greater standardization. However, there are challenges such as low response rate, the exclusion of individuals with low education, superficial responses and inadequacies to the context, which can compromise the representativeness of the sample and of the analyses ^66^.

Given this context, participatory and collaborative approaches, using instruments, can provide a more detailed view of the impacts and needs of affected populations. They overcome the limitations of traditional methods, which often do not sufficiently involve affected communities, enabling a more complete and shared understanding of disasters ^53^.

The choice of binary elements in the reviewed studies allows respondents to choose between two options, facilitating reflection before answering. In contrast, Likert-type scales provide variations in the intensity of responses, offering a more detailed analysis. However, scales with more points require greater analysis and can be challenging for respondents with lower educational level or higher cognitive load ^67^ ^,^ ^68^ ^,^ ^69^.

The use of free response patterns in interviews favors the interaction between researcher and participant, allowing access to subjective information with common sense language and free answers ^70^. It is noted, however, that the limitations for this type of instrumental approach, associated with individuals’ own reasons in providing the answers or the influence of the personal aspect of the applying researcher, among other disadvantages that compromise the perspective on the analyzed situation ^70^.

## Final considerations

The reviewed articles used different instruments to assess exposure to oil spills and health impacts, mainly mental and psychological impacts, perception of environmental risks and damages, in addition to effects on socioeconomic aspects and social cohesion issues. Although these instruments provide an expanded view of the events, there is a gap in studies with an integrated approach to the environmental and socioeconomic dimensions of the impacts. Future research should expand the analysis on the consistency, reliability and cross-cultural adaptations of the instruments. These pieces of information can help create a multidimensional matrix for monitoring disasters, facilitating quick and effective decisions in designing protection and recovery policies.
