# Supplementary material for: Instruments for assessing the impacts of oil spills: an integrated approach to health, the environment and the socioeconomic profile of exposed areas
Source: Cad Saude Publica. 2025 Mar 31;41(2):e00228723. doi: 10.1590/0102-311XEN228723 (PMC11960760; doi:10.1590/0102-311XEN228723)
Supplement: Translation

# Translation: Instrumentos de avaliação dos impactos de derramamentos de petróleo: abordagem integrada na saúde, no ambiente e no perfil socioeconômico de áreas expostas

**DOI:** 10.1590/0102-311XPT228723

**Authors:** Loic Hernandez do Amaral e Aragão, Maria Juliana Ferreira dos Santos, Aline do Monte Gurgel, Mariana Olivia Santana dos Santos, Mariana Maciel Nepomuceno, Idê Gomes Dantas Gurgel

**Affiliations:** 1 Instituto Aggeu Magalhães, Fundação Oswaldo Cruz, Recife, Brasil.; 2 Faculdade de Ciências da Saúde, Universidade Federal do Rio Grande do Norte, Santa Cruz, Brasil.; 3 Centro Acadêmico do Agreste, Universidade Federal de Pernambuco, Caruaru, Brasil.; 4 Faculdade Pernambucana de Saúde, Recife, Brasil.

**Keywords:** Poluição por Petróleo, Inquéritos e Questionários, Avaliação de Desastres

## Abstract

Os avanços da indústria de petróleo estiveram associados a grandes desastres envolvendo derramamento do material em campos marítimos, impactando negativamente a vida e o ambiente. Considera-se a importância do acompanhamento e da avaliação desses eventos, a partir de instrumentos variados, sobre três eixos de investigação: a saúde; o ambiente; e a situação socioeconômica das populações expostas. Desse modo, pretendeu-se mapear, por meio de uma revisão de escopo, as evidências científicas envolvendo a aplicação desses instrumentos sobre os impactos de derramamentos de petróleo. Foram utilizadas diferentes bases de dados e idiomas para a busca dos trabalhos. Os dados foram revisados por dupla de pesquisadores, que realizaram a avaliação qualitativa. Para a síntese dos resultados, foram considerados 45 estudos distribuídos entre os tipos observacionais sem grupo controle, de coorte com grupo controle e transversais, com predomínio daqueles voltados para o eixo saúde (n = 39; 86,66%) e com método de entrevista (n = 29; 64,44%). Foram identificados 75 registros de instrumentos utilizados, com escalas do tipo Likert, escalas combinadas e padrões de respostas livres. Ademais, observou-se a carência de estudos com investigação nos eixos ambiental e socioeconômico, sobretudo de forma integrada. Ao fim, considera-se a importância de novas pesquisas que incluam características essenciais dos instrumentos (consistência, confiabilidade, fidedignidade, adaptações transculturais) para a possibilidade de construir matrizes multidimensionais de acompanhamento de desastres motivados pela ação humana, facilitando tomadas de decisões na elaboração de políticas e ações governamentais.

## Introdução

Historicamente, o desenvolvimento industrial e a crescente demanda por energia têm impulsionado o crescimento econômico global. Nesse contexto, a indústria de petróleo, que se originou no século XIX nos Estados Unidos, destacou-se como uma das principais fontes de energia mundial ^1^ ^,^ ^2^ ^,^ ^3^. Além disso, o crescimento econômico de diversas nações passou a depender fortemente de combustíveis fósseis, como petróleo, gás natural e carvão, que movimentam diretamente a economia global e o comércio internacional ^4^.

As principais reservas de petróleo se encontram em campos marítimos profundos, cuja exploração exige avanços tecnológicos constantes. Esses avanços são essenciais para a extração, o transporte e o refino, mas também enfrentam desafios geológicos, climáticos e operacionais, que podem resultar em grandes danos humanos, materiais e ambientais quando não superados adequadamente ^3^ ^,^ ^5^ ^,^ ^6^ ^,^ ^7^. Assim, é importante ressaltar que a exploração de petróleo está entre as atividades humanas mais poluentes, pois gera enormes volumes de resíduos sólidos e gases nocivos que degradam o meio ambiente e prejudicam a saúde humana ^8^.

Nesse sentido, a segurança ao longo da cadeia produtiva é crucial para evitar acidentes de grandes proporções, como derramamentos de petróleo, que causam sérios danos ambientais e riscos à saúde dos seres vivos, tornando-os vulneráveis ao aumento da incidência de câncer, de doenças neurológicas e psíquicas, tegumentares, hepáticas, cardiovasculares e respiratórias ^9^ ^,^ ^10^.

Não por acaso, ao longo da história, vários desastres envolvendo petróleo demonstraram o impacto devastador dessas ocorrências. O primeiro grande caso registrado foi o desastre do Sinclair Petrolore (1960), seguido por outros como o Torrey Canyon (1967), Exxon Valdez (1989), Hebei Spirit (2007) e, mais recentemente, o Deepwater Horizon (2010) ^11^ ^,^ ^12^ ^,^ ^13^ ^,^ ^14^ ^,^ ^15^ ^,^ ^16^ ^,^ ^17^ ^,^ ^18^ ^,^ ^19^. Cada um desses desastres trouxe consequências severas, tanto para os ecossistemas atingidos quanto para as populações locais, expondo-as a sérios problemas de saúde e perdas econômicas.

No Brasil, o desastre de 2019 evidenciou a gravidade de derramamentos de óleo. As manchas de petróleo que surgiram no litoral nordestino afetaram mais de 3.400km de costa, impactando diretamente o ecossistema, a economia local e a saúde pública. Além disso, o derramamento continua gerando desafios ambientais, uma vez que fragmentos de petróleo continuam a surgir nas praias, sem que a origem do problema tenha sido identificada ^20^ ^,^ ^21^.

Diante desses desastres, a utilização de instrumentos de avaliação e monitoramento tem se mostrado fundamental para lidar com os impactos gerados. Tais instrumentos - que incluem escalas, questionários e *checklists* - são utilizados para medir as consequências dos derramamentos e subsidiar a tomada de decisões em situações de emergência ^22^ ^,^ ^23^. Contudo, a escolha adequada desses instrumentos é vital, pois a diversidade de variáveis envolvidas pode influenciar diretamente a confiabilidade dos resultados ^24^.

Considerando a carência de estudos que correlacionem a aplicação desses instrumentos em contextos de derramamentos de petróleo, pressupõe-se que, ao identificá-los, o acompanhamento e a resposta, bem como a intervenção sobre a situação, tornam-se mais ágeis e eficazes, permitindo uma melhor condução das ações em diferentes abordagens, sejam elas direcionadas aos aspectos de cuidados em saúde, de proteção e recuperação ambiental, ou de reparos socioeconômicos.

Assim, o presente estudo visa mapear as evidências científicas relacionadas à aplicação desses instrumentos para avaliar, acompanhar e monitorar os impactos de derramamentos de petróleo na saúde, no ambiente e no perfil socioeconômico das áreas afetadas.

## Método

Trata-se de um estudo do tipo revisão de escopo (*scoping review*), utilizado no mapeamento de uma lacuna sobre um campo de investigação de interesse, e considerado um estágio preliminar à realização de uma revisão sistemática com rigor metodológico e reprodutibilidade, em termos de natureza, características e volume ^25^ ^,^ ^26^ ^,^ ^27^.

Para essa abordagem, adotou-se o guia internacional *Preferred Reporting Items for Systematic Reviews and Meta-Analyses Extension for Scoping Reviews* (PRISMA-ScR) e a recomendação proposta pelo Instituto Joanna Briggs, amplamente difundidos e adotados por diversos estudos que utilizam a metodologia da revisão de escopo em seis etapas: (i) definição da questão; (ii) identificação dos estudos; (iii) seleção dos estudos com base nos critérios de inclusão e exclusão; (iv) extração de dados; (v) organização dos resultados; e (vi) divulgação dos relatórios da pesquisa ^28^ ^,^ ^29^ ^,^ ^30^.

Este estudo foi registrado no Open Science Framework (https://osf.io/y792v/), com o objetivo de armazenar e fornecer a transparência quanto ao protocolo utilizado. Além disso, quanto à questão norteadora, utilizou-se o método População-Conceito-Contexto (PCC) para a identificação dos tópicos-chave, como segue: como os instrumentos de medida e acompanhamento dos impactos de derramamentos de petróleo são utilizados para a avaliação da saúde, do ambiente e do perfil socioeconômico de populações e áreas expostas?

Foram utilizadas, para fins deste estudo, as seguintes bases de dados: MEDLINE via PubMed, Cochrane Library, Biblioteca Virtual em Saúde (BVS), Portal de Periódicos da CAPES, Scopus, Web of Science, Embase e SciELO. Todas as bases apresentam o escopo de estudos que atendem ao objetivo e à questão norteadora apresentados. Ademais, não foram consideradas as fontes de literatura cinzenta, as quais não foram publicadas formalmente.

A busca bibliográfica para o desenvolvimento deste estudo foi realizada por meio da relação de termos pertinentes ao objeto central do estudo, com operadores booleanos AND e OR, filtrando os resultados nas diferentes bases de dados. Os termos utilizados, assim como a estratégia de busca, foram regulados pelo *Medical Subject Headings* (MeSH): *petroleum pollution*, *surveys and questionnaires* e seus respectivos *entry terms*, conforme Quadro 1.

**Quadro 1:** Estratégias de busca aplicadas por base de dados utilizando operadores AND e OR.

BASE DE DADOS	ESTRATÉGIA DE BUSCA
PubMed	((((((((((((Petroleum Pollution[Title/Abstract]) OR (Petroleum Pollutions[Title/Abstract])) OR (Pollution, Petroleum[Title/Abstract])) OR (Pollutions, Petroleum[Title/Abstract])) OR (Oil Spills[Title/Abstract])) OR (Oil Spill[Title/Abstract])) OR (Spill, Oil[Title/Abstract])) OR (Spills, Oil[Title/Abstract])) OR (Oil Pollution[Title/Abstract])) OR (Oil Pollutions[Title/Abstract])) OR (Pollution, Oil[Title/Abstract])) OR (Pollutions, Oil[Title/Abstract])) AND ((((((((((((((((((((((((((((((((Surveys[Title/Abstract] AND Questionnaires[Title/Abstract]) OR (Questionnaires[Title/Abstract] AND Surveys[Title/Abstract])) OR (Survey Methods[Title/Abstract])) OR (Methods, Survey[Title/Abstract])) OR (Survey Method[Title/Abstract])) OR (Methodology, Survey[Title/Abstract])) OR (Survey Methodology[Title/Abstract])) OR (Community Surveys[Title/Abstract])) OR (Survey[Title/Abstract])) OR (Surveys[Title/Abstract])) OR (Repeated Rounds of Survey[Title/Abstract])) OR (Surveys, Community[Title/Abstract])) OR (Survey, Community[Title/Abstract])) OR (Respondent[Title/Abstract])) OR (Respondents[Title/Abstract])) OR (Surveys, Baseline[Title/Abstract])) OR (Survey, Baseline[Title/Abstract])) OR (Baseline Surveys[Title/Abstract])) OR (Baseline Survey[Title/Abstract])) OR (Questionnaire Designs[Title/Abstract])) OR (Designs, Questionnaire[Title/Abstract])) OR (Design, Questionnaire[Title/Abstract])) OR (Questionnaire Design[Title/Abstract])) OR (Nonrespondent[Title/Abstract])) OR (Nonrespondents[Title/Abstract])) OR (Questionnaire[Title/Abstract])) OR (Questionnaires[Title/Abstract])) OR (Techniques, Randomized Response[Title/Abstract])) OR (Response Techniques, Randomized[Title/Abstract])) OR (Response Technique, Randomized[Title/Abstract])) OR (Randomized Response Techniques[Title/Abstract])) OR (Randomized Response Technique[Title/Abstract]))
Cochrane Library	(Petroleum Pollution) OR (Spills, Oil) OR (Pollutions, Oil) OR (Oil Spills) OR (Oil Pollutions) OR (Oil Spills Effects) in Title Abstract Keyword AND (Surveys and Questionnaires) OR (Questionnaire Designs) OR (Baseline Surveys) OR (Methods, Survey) AND (Questionnaires) in Title Abstract Keyword - (Word variations have been searched)
Biblioteca Virtual em Saúde (BVS)	(petroleum pollution) OR (oil spill) OR (oil pollution) OR (oil spills effects) AND (surveys and questionnaires) OR (questionnaires) OR (questionnaire designs ) OR (baseline surveys) OR (methods, survey)
Web of Science	(((AB=(oil spills)) OR AB=(oil spills effects)) OR AB=(petroleum pollution)) OR AB=(oil pollution) AND ((((AB=(surveys and questionnaires)) OR AB=(questionnaires)) OR AB=(questionnaire designs )) OR AB=(baseline surveys)) OR AB=(methods, survey)
Embase	(‘petroleum pollution’/exp OR ‘petroleum pollution’ OR ((‘petroleum’/exp OR petroleum) AND (‘pollution’/exp OR pollution)) OR ‘oil spill’:ti,ab,kw OR ‘oil spill effects’:ti,ab,kw OR ‘oil pollution’:ti,ab,kw) AND ([controlled clinical trial]/lim OR [randomized controlled trial]/lim) AND [2011-2023]/py AND ((‘surveys’/exp OR surveys) AND (‘questionnaires’/exp OR questionnaires) OR questionnaire:ti,ab,kw OR ‘questionnaire designs’:ti,ab,kw OR ‘methods, survey’:ti,ab,kw) AND ([controlled clinical trial]/lim OR [randomized controlled trial]/lim) AND [2011-2023]/py
SciELO	(ti:(petroleum pollution)) OR (ti:(oil spill)) OR (ti:(oil spills effects)) OR (ti:(oil pollution)) AND (ti:(surveys and questionnaires)) OR (ti:(surveys)) OR (ti:(questionnaires)) OR (ti:(questionnaire design)) OR (ti:(baseline survey)) OR (ti:(nonrespondent)) OR (ti:(randomized response technique)) OR (ti:(survey methods))
Scopus	(petroleum pollution) OR (oil spill) OR (oil pollution) OR (oil spills effects) AND (surveys and questionnaires) OR (questionnaire) OR (questionnaire designs ) OR (baseline surveys) OR (methods, survey) OR (nonrespondent)

Fonte: elaboração própria.

Foram identificados 2.055 registros nas bases de dados. Após eliminar 167 duplicados, restaram 1.888 para leitura de títulos e resumos. Desses, 1.780 foram excluídos por falta de informações relevantes, resultando em 108 registros para leitura completa. Finalmente, 45 registros foram selecionados para a revisão e síntese narrativa (Figura 1).

**Figura 1:** Fluxograma indicando o processo de seleção dos estudos adaptado do *Preferred Reporting Items for Systematic Reviews and Meta-Analyses Extension for Scoping Reviews* (PRISMA-ScR).

Foram incluídos todos os estudos originais nos idiomas inglês, português e espanhol, publicados e disponíveis integralmente, que contemplam diferentes metodologias, devendo responder à questão norteadora de modo a atender à correlação entre a exposição à desastres com derramamento de petróleo e os impactos decorrentes dessa, utilizando-se instrumentos de mensuração dos efeitos.

Os estudos selecionados, publicados entre 2011 e 2023, refletem o aumento significativo de pesquisas sobre derramamentos de petróleo após a explosão da plataforma Deepwater Horizon em 2010, o segundo maior desastre de petróleo da história e o maior em ambiente aquático ^31^.

Foram excluídos estudos de dados secundários, revisões, casos únicos ou séries de casos, relatos, opiniões de especialistas, editoriais e similares. Também foram excluídos trabalhos acadêmicos (teses e dissertações), livros, capítulos de livros, anais de eventos, relatórios e documentos. Além disso, foram removidos estudos duplicados ou irrelevantes conforme a leitura de títulos, resumos ou textos completos.

Para a etapa de seleção dos estudos, dois revisores independentes tiveram auxílio da plataforma Rayyan QCR (https://www.rayyan.ai/) nas etapas de leitura dos títulos e resumos e de leitura do texto na íntegra. Em caso de discordância, um terceiro revisor independente seria necessário para decisão final. Na etapa seguinte, as informações extraídas foram exportadas para o software Microsoft Excel (https://products.office.com/), obedecendo-se a caracterização dos estudos selecionados para a extração da amostra (autor[es], periódico, local de publicação, objetivos, tipo de estudo, população, eixo de investigação, principais resultados, pesquisa de formação preliminar, e nível de evidência), assim como a caracterização dos instrumentos utilizados nos estudos (identificação, aplicação do instrumento pelo estudo, dimensões analisadas, informações adicionais e padrões de respostas aos instrumentos utilizados).

O nível de evidência utilizado para caracterização dos estudos também seguiu as recomendações do Instituto Joanna Briggs em cinco níveis e suas subclassificações ^32^, com escala do nível de maior recomendação (1) para estudos do tipo experimentais até o nível de menor recomendação (5) para opiniões de especialistas e pesquisas de bancada, esse último não sendo aplicável para o presente estudo, obedecendo-se o critérios de exclusão adotados.

Os dados extraídos foram apresentados em quadros, analisados sob estatística descritiva (frequência absoluta e relativa), seguidos de síntese narrativa para a construção dos resultados deste estudo. Por fim, por se tratar de um estudo produzido a partir de dados secundários, de livre acesso, sem exposição direta de seres humanos e/ou animais para a coleta dos dados, não precisou ser submetido à apreciação de Comitê de Ética em Pesquisa competente.

Este estudo conta com o apoio financeiro do Programa Pesquisa para o Sistema Único de Saúde: Gestão Compartilhada em Saúde - PPSUS/Pernambuco (Conselho Nacional de Desenvolvimento Científico e Tecnológico, Departamento de Ciência e Tecnologia da Secretaria de Ciência, Tecnologia e Inovação do Ministério da Saúde, Secretaria de Estado da Saúde de Pernambuco, Fundação de Amparo à Ciência e Tecnologia de Pernambuco [CNPq/Decit/SCTIE/MS/SES/Facepe]), do Programa Inova Fiocruz - *Encomendas Estratégicas: Territórios Sustentáveis e Saudáveis no Contexto da Pandemia de COVID-19* e do edital para projetos e ações estratégicas territorializadas para a implementação da Agenda 2030, da Presidência da Fundação Oswaldo Cruz (FIOCRUZ), e da Coordenação de Aperfeiçoamento de Pessoal de Nível Superior (CAPES; Código Financeiro 001).

## Resultados

Quanto à caracterização dos registros selecionados (Quadro 2), observou-se que a maioria dos estudos foi realizada nos Estados Unidos (71,11%), com menor frequência na Coreia do Sul (13,33%), Nigéria (6,66%), Espanha (4,44%), Reino Unido (2,22%) e Brasil (2,22%). O maior volume de publicações foi em 2019 (20%), seguido por 2022 (15,55%) e 2012 (11,11%). Apenas um estudo foi publicado em 2023, por pesquisadores dos Estados Unidos.

**Quadro 2:** Caracterização dos estudos selecionados.

ESTUDO (ANO)	LOCAL	OBJETIVOS	TIPO DE ESTUDO POPULAÇÃO NÍVEL DE EVIDÊNCIA *	EIXO	PRINCIPAIS RESULTADOS	PESQUISA DE FORMAÇÃO
Denic-Roberts et al. ^71^ (2023)	Estados Unidos	Investigar os riscos de condições neurológicas de longo prazo entre os respondentes da Guarda Costeira dos Estados Unidos ao derramamento de óleo do Deepwater Horizon em 2010	Coorte com grupo controle 5.964 respondentes e 39.260 não-respondentes 3.c	Saúde	Observou-se aumento moderado a alto dos riscos de desenvolvimento de condições neurológicas em longo prazo, incluindo-se dores de cabeça, enxaqueca, zumbido e condições inflamatórias de nervos	NI
Denic-Roberts et al. ^72^ (2022)	Estados Unidos	Investigar os sintomas e as condições cardiovasculares prevalentes e incidentes na coorte da Guarda Costeira exposta ao derramamento de óleo do Deepwater Horizon	Coorte com grupo controle 5.964 respondentes e 39.260 não-respondentes 3.c	Saúde	Maiores níveis de exposição estiveram associados a maior prevalência de dor no peito, além de elevado risco de distúrbios de valvula mitral, palpitações e hipertensão arterial	NI
Goldman et al. ^73^ (2022)	Estados Unidos	Avaliar a associação entre exposição ao derramamento de óleo do Deepwater Horizon, a capacidade de enfrentamento/apoio emocional e a gravidade dos sintomas de ansiedade	Transversal 38.361 indivíduos 4.b	Saúde	O contato direto com o óleo esteve associados a maior incidência de aumento de sintomas de ansiedade	NI
Ferreira et al. ^74^ (2022)	Brasil	Descrever e estimar os impactos do derramamento de óleo sobre variáveis sociais, econômicas e de saúde das principais populações afetadas no litoral do Nordeste do Brasil	Observacional sem grupo controle 19.300 indivíduos em cidades pesqueiras; 40.056 indivíduos em cidades 3.e	Saúde; socioeconômico	Observou-se impactos heterogêneos entre os indicadores socioeconômicos dos segmentos e municípios avaliados, com as regiões de turismo e pesca sendo a mais afetadas em termos de redução de renda e venda de produtos. Além disso, os indicadores de saúde apontam sintomas de intoxicação exógena como principal dano	NI
Kwok et al. ^75^ (2022)	Estados Unidos	Determinar se a participação em atividades de limpeza após o desastre do Deepwater Horizon foi associada a um risco aumentado de desenvolver hipertensão	Coorte com grupo controle 6.846 respondentes e 1.505 não-respondentes 3.c	Saúde	Observou-se que maiores níveis de exposição estavam associadas a maior risco de desenvolvimento de hipertensão	NI
Lawrence et al. ^76^ (2022)	Estados Unidos	Avaliar os riscos primários de inalação de produtos químicos para a limpeza de óleo experimentados pelos trabalhadores das respostas de contenção após o desastre do Deepwater Horizon	Coorte com grupo controle 19.018 respondentes e 5.585 não-respondentes 3.c	Saúde	A exposição aos produtos químicos esteve associada ao aumento do risco de asma	NI
Oghenetega et al. ^77^ (2022)	Nigéria	Determinar o efeito da exposição materna à poluição por óleo sobre os resultados maternos na região do Delta do Níger (Nigéria)	Coorte com grupo controle 1.720 mulheres grávidas entre áreas de alta e baixa exposição 3.c	Saúde	Observou-se que mulheres em áreas com alta exposição à poluição por óleo têm um risco maior de hemorragia pós-parto e ruptura prematura de membrana em comparação com mulheres em áreas de baixa exposição	NI
Rusiecki et al. ^78^ (2022)	Estados Unidos	Avaliar as condições respiratórias incidentes associadas à resposta ao derramamento de óleo do Deepwater Horizon	Coorte com grupo controle 5.964 respondentes e 39.260 não-respondentes 3.c	Saúde	Maiores exposições estiveram associadas a maior incidência de sinusite, doença pulmonar obstrutiva crônica, dispneia e condições relacionadas	NI
Choi et al. ^15^ (2021)	Coreia do Sul	Avaliar a prevalência e o risco de sintomas psicológicos ao longo de 9 anos desde o derramamento de óleo do Hebei Spirit	Coorte com grupo controle 2.013 indivíduos em área de alta exposição e 6.495 em área de baixa exposição 3.c	Saúde	Observou-se uma associação significativa entre o maior tempo de trabalho de limpeza em indivíduos com menor renda familiar e baixa escolaridade e o risco para depressão, ansiedade e TEPT	NI
Eleke et al. ^79^ (2021)	Nigéria	Examinar o efeito da poluição ambiental por petróleo bruto nos resultados do nascimento de recém-nascidos em Rivers State (Nigéria)	Coorte com grupo controle 169 registros em uma área afetada e 169 registros em uma área não afetada 3.c	Saúde	Aumento dos riscos de parto prematuro, crescimento mais lento e morbidade neonatal dentro de 6 semanas após o nascimento em ambientes mais afetados por petróleo	NI
Harville et al. ^80^ (2021)	Estados Unidos	Examinar a relação entre a exposição ao derramamento de óleo e os resultados de nascimento perto do Golfo do México	Observacional sem grupo controle 1.375 mulheres, sendo 503 que tiveram filhos antes e depois do derramamento 3.e	Saúde	As associações entre a maioria dos indicadores de exposição a derramamento de óleo e os resultados da gravidez foram nulas, embora com altos níveis de contato com óleo	NI
Bergstrand & Mayer ^81^ (2020)	Estados Unidos	Investigar as percepções de longo prazo da recuperação da comunidade após o derramento de óleo	Coorte com grupo controle 351 indivíduos em áreas com alta e baixa exposição ao derramamento 3.c	Ambiental; saúde; socioeconômico	Observou-se a influência da comunidade na percepção de recuperação após o desastre, embora os relatos apontem para a percepção de pouca recuperação econômica e ambiental	NI
Bebeteidoh et al. ^82^ (2020)	Reino Unido	Determinar o impacto das atividades das refinarias locais de petróleo bruto no Delta do Níger (Nigéria) sobre suas comunidades anfitriãs	Transversal 487 indivíduos 4.b	Ambiental	Observou-se impactos em rotas de pesca e áreas de cultivo, afetando a fonte de subsistência de trabalhadores da pesca e da agricultura	NI
Oghenetega et al. ^83^ (2020)	Nigéria	Determinar a associação entre poluição por óleo e aborto espontâneo, natimorto e morte infantil na região do Delta do Níger (Nigéria)	Coorte com grupo controle 782 mulheres em área de alta exposição e 782 mulheres área de baixa exposição 3.c	Saúde	Maior incidência de morte infantil na região de alta exposição à poluição por óleo, sem associação entre alta exposição e aborto espontâneo e natimorto	NI
Parker et al. ^84^ (2020)	Estados Unidos	Examinar a natureza e os preditores de preocupação com os impactos contínuos do derramamento de óleo do Deepwater Horizon em 2010	Observacional sem grupo controle 903 respondentes de uma amostra de 2.520 indivíduos 3.e	Saúde; socioeconômico	Maior exposição ao derramamento de óleo esteve associada a níveis mais altos de preocupação com os impactos, sobretudo aqueles relacionados à saúde	NI
Erickson et al. ^85^ (2019)	Estados Unidos	Avaliar a relação entre a exposição ao calor ambiental e os sintomas relacionados entre os respondentes do desastre com o Deepwater Horizon	Transversal 3.648 indivíduos 4.b	Saúde	Maiores exposições ao calor durante as ações de resposta ao derramamento estiveram associadas à maior prevalência de sintomas relacionados ao calor em comparação com aqueles com menor exposição	NI
Kaufman et al. ^86^ (2019)	Estados Unidos	Avaliar a associação entre o contato direto com o óleo e a gravidade da depressão entre os residentes da costa do Golfo do México após o derramamento de óleo da Deepwater Horizon e avaliar a potencial moderação dessa associação por participação de limpeza, autodomínio ou apoio emocional	Transversal 38.361 indivíduos 4.b	Saúde	Observou-se que o contato com o óleo foi associado ao aumento da gravidade da depressão, sobretudo para aqueles com menor autodomínio ou apoio emocional	NI
Krishnamurthy et al. ^87^ (2019)	Estados Unidos	Avaliar a associação entre exposições a petróleo bruto e sintomas neurológicos agudos relatados por respondentes do derramamento do Deepwater Horizon	Coorte com grupo controle 4.855 respondentes e 44.823 não-respondentes 3.c	Saúde	A exposição isolada ao petróleo ou combinada com os produtos químicos esteve moderada/fortemente associada ao aumento da prevalência de sintomas neurológicos agudos	NI
Nugent et al. ^88^ (2019)	Estados Unidos	Descrever os perfis de TEPT entre as mulheres e a associação ao nível de exposição ao derramamento de óleo do Deepwater Horizon	Observacional sem grupo controle 1.997 mulheres de uma amostra inicial de 2.852 3.e	Saúde	Observou-se 5 perfis de mulheres com TEPT, variando entre níveis mais brandos e mais graves de sintomas, associados ao grau de exposição	NI
Quist et al. ^19^ (2019)	Estados Unidos	Examinar a associação de concentrações de THC e classes de trabalho de contenção ao derramamento do Deepwater Horizon com a função neurocomportamental entre trabalhadores	Observacional sem grupo controle 3.291 respondentes de uma amostra total de 32.608 indivíduos 3.e	Saúde	Trabalhadores mais expostos apresentaram maior alteração nos quesitos de atenção, memória e função executiva	NI
Rung et al. ^89^ (2019)	Estados Unidos	Descrever as mudanças na saúde mental entre as mulheres após o derramamento de óleo e examinar sua relação com a exposição ao longo do tempo	Observacional sem grupo controle 2.038 mulheres de uma amostra inicial de 2.852 3.e	Saúde	Observou-se que os sintomas depressivos aumentaram após o derramamento de óleo e que a associação entre exposição econômica e física persistiram até 6 anos após o desastre	NI
Strelitz et al. ^90^ (2019)	Estados Unidos	Avaliar as associações entre a duração do trabalho de limpeza do derramamento de óleo, a proximidade residencial do derramamento de óleo e a incidência de infarto do miocárdio autorrelatado ou doença coronariana fatal	Observacional sem grupo controle 21.256 indivíduos de uma amostra inicial de 32.608 3.e	Saúde	Morar próximo ao derramamento de óleo (*vs.* morar mais longe) foi associado a doenças cardíacas, assim como a maior duração de trabalho esteve associada ao aumento do risco, persistindo por 5 anos	NI
Strelitz et al. ^91^ (2019)	Estados Unidos	Avaliar a relação entre a exposição ao THC usado durante a resposta e limpeza do derramamento de óleo Deepwater Horizon e o risco de infarto agudo de miocárdio	Observacional sem grupo controle 16.814 indivíduos de uma amostra inicial de 24.375 3.e	Saúde	Maiores níveis de exposição ao químico estiveram associadas a maior risco de infarto agudo de miocárdio, com registros de 312 incidentes	NI
Werder et al. ^92^ (2019)	Estados Unidos	Avaliar as associações entre os níveis de BTEX no sangue e sintomas em residentes da costa do Golfo do México	Observacional sem grupo controle 690 indivíduos de uma amostra inicial de 1.055 3.e	Saúde	Observou-se que metade dos indivíduos apresentou pelo menos um sintoma neurológico após exposição ao produto químico	NI
Alexander et al. ^93^ (2018)	Estados Unidos	Examinar a associação entre exposições específicas encontradas durante a limpeza de derramamento de óleo e sintomas respiratórios agudos	Transversal 4.855 socorristas envolvidos nos esforços de contenção ao derramamento 4.b	Saúde	Maior prevalência de tosse, seguida de falta de ar e “chiado no peito”, sugerindo uma correlação entre exposição e efeito	NI
Harville et al. ^94^ (2018)	Estados Unidos	Examinar a associação entre a exposição autorrelatada ao derramamento de petróleo e o autorrelato de aborto espontâneo ou infertilidade	Observacional sem grupo controle 1.620 mulheres, incluindo 443 que estavam grávidas durante a entrevista 3.e	Saúde	Observou-se um risco aumentado de aborto espontâneo para qualquer nível de exposição ao óleo e problemas de fertilidade nas mulheres mais expostas ao derramamento	NI
Rusiecki et al. ^95^ (2018)	Estados Unidos	Investigar os potenciais efeitos agudos e de longo prazo na saúde decorrentes de exposições de trabalho de resposta a derramamentos de óleo	Coorte com grupo controle 8.696 respondentes e 44.823 não-respondentes 3.c	Saúde	A exposição ao petróleo bruto esteve associada a sintomas relacionados a tosse, falta de ar, coceiras, dores de cabeça, tontura, diarreias, dor no estômago, nausea/vômito, ardência ao urinar e asma	NI
Strelitz et al. ^96^ (2018)	Estados Unidos	Avaliar a relação do trabalho de limpeza e a proximidade com o derramamento de óleo com o risco autorrelatado de infarto do miocárdio	Observacional sem grupo controle 31.109 indivíduos sem história previa de infarto do miocárdio 3.e	Saúde	Foram registrados 192 infartos durante o período do estudo; 151 entre os trabalhadores. O trabalho de limpeza e a proximidade foram sugestivamente associados a um possível risco aumentado de infarto do miocárdio não fatal	NI
Croisant et al. ^17^ (2017)	Estados Unidos	Compreender os efeitos na saúde física e na saúde mental atribuíveis ao derramamento de óleo de Macondo	Transversal 324 indivíduos 4.b	Saúde	Observou-se mudanças no estado de saúde física e mental autorrelatado após o derramamento de óleo, disparidades no acesso aos cuidados de saúde e associações entre saúde mental e condições emocionais relacionadas ao deslocamento/desemprego	NI
Harville et al. ^18^ (2017)	Estados Unidos	Examinar a associação entre a exposição autorrelatada aos efeitos físicos, sociais e econômicos do derramamento de óleo no Golfo do México e complicações na gravidez	Observacional sem grupo controle 1.650 mulheres, incluindo 460 grávidas durante a entrevista 3.e	Saúde; socioeconômico	Não foi observada associação entre exposição ao derramamento e distúrbios hipertensivos, no entanto, houve maior propensão ao diabetes gestacional	NI
Kwok et al. ^97^ (2017)	Estados Unidos	Analisar os efeitos do desastre do Deepwater Horizon na saúde mental de indivíduos envolvidos na resposta e limpeza de derramamento de óleo	Coorte com grupo controle 8.968 respondentes e 2.225 não respondentes 3.c	Saúde	Observou-se aumento da prevalência de depressão naqueles indivíduos envolvidos no trabalho de limpeza	NI
McGowan et al. ^98^ (2017)	Estados Unidos	Examinar as associações entre a exposição potencial aos dispersantes e sintomas adversos respiratórios, dérmicos e de irritação ocular	Observacional sem grupo controle Entre 27.659 e 29.468 participantes que participaram da entrevista 3.e	Saúde	Houve associação significative entre a exposição potencial a qualquer um dos dispersantes e todos os resultados de saúde, destacando-se a queimação no nariz, garganta ou pulmões, aperto no peito e queimação nos olhos	NI
Zilversmit et al. ^99^ (2017)	Estados Unidos	Comparar frutos do mar com os níveis sanguíneos de Hg e n-3 PUFAs entre mulheres grávidas e não grávidas	Observacional sem grupo controle 634 mulheres dentre 1.788 recrutadas 3.e	Saúde	Observou-se maiores níveis de Hg no sangue e nos frutos do mar de mulheres grávidas, além de mudanças no comportamento alimentar, que apresentou redução do consume geral de peixes	NI
Nriagu et al. ^100^ (2016)	Estados Unidos	Determinar a prevalência e os correlatos de medidas de saúde e sofrimento emocional em uma área do Delta do Níger (Nigéria), explorar a percepção dos riscos ambientais pela população local e sua influência no sofrimento emocional e estabelecer relações entre a exposição ao petróleo, poluição e as medidas de resultados de saúde	Transversal 600 indivíduos 4.b	Ambiental; saúde	Alto nível de sofrimento foi observado em toda a população estudada. A percepção de risco foi, em grande medida, por perigos temidos, pistas visuais, e pistas quimiossensoriais. As métricas de exposição foram consideradas preditores significativos dos efeitos na saúde e fatores influenciadores (reações emocionais)	NI
Peres et al. ^101^ (2016)	Estados Unidos	Caracterizar a exposição individual ao derramamento do Deepwater Horizon e examinar a sua associação com a saúde física	Observacional sem grupo controle 2.852 mulheres de uma amostra inicial de 42.649 3.e	Saúde	Observou-se a relação significative entre a alta exposição físico-ambiental e todos os sintomas de saúde física, com associações mais fortes para queimação no nariz, garganta ou pulmões, dor de garganta, tontura e chiado	NI
Rung et al. ^102^ (2016)	Estados Unidos	Descrever a relação entre exposição a derramamento de óleo e a saúde mental entre mulheres que vivem na região costeira do sul da Louisiana (Estados Unidos)	Observacional sem grupo controle 2.842 mulheres 3.e	Saúde	Observou-se que as exposições econômica e física foram significativamente associadas a sintomas depressivos e conflitos, enquanto apenas a exposição física foi associada ao sofrimento mental	NI
Simon-Friedt et al. ^103^ (2016)	Estados Unidos	Determinar riscos percebidos dentro de comunidades expostas ao derramamento do Deepwater Horizon	Observacional sem grupo controle 192 mulheres 3.e	Ambiental	Observou-se a redução significativa do consumo de frutos do mar, associada a percepções ambientais negativas que permancem ao longo do tempo	NI
Ha et al. ^104^ (2013)	Coreia do Sul	Examinar a saúde mental das crianças na área afetada pelo acidente de derramamento de óleo Hebei Spirit	Transversal 1.362 crianças em idade escolar na região 4.b	Saúde	Houve relação significativa entre a maior proximidade com a região afetada e o risco de sintomas de depressão, sem associação para a ansiedade	NI
Jung et al. ^105^ (2013)	Coreia do Sul	Avaliar o efeito respiratório da exposição ao derramamento de óleo em crianças da Costa Amarela (Coreia do Sul)	Observacional sem grupo controle 436 crianças de uma amostra inicial de 662 3.e	Saúde	As crianças que viviam perto da área do derramamento de óleo apresentaram redução da função pulmonar, uma prevalência aumentado de asma e hiperresponsividade	NI
Buttke et al. ^106^ (2012)	Estados Unidos	Determinar as necessidades gerais e de saúde mental da comunidade um ano após o derramamento de óleo Deepwater Horizon	Transversal Amostragem de domicílios 4.b	Saúde	Os entrevistados que relataram diminuição da renda após o derramamento de óleo eram mais propensos a relatar sintomas de saúde mental	NI
Gwack et al. ^107^ (2012)	Coreia do Sul	Investigar os efeitos agudos na saúde e seus fatores relacionados entre os militares que participaram da limpeza do derramamento de óleo do Hebei Spirit	Coorte com grupo controle 2.050 respondentes e 574 não-respondentes 3.c	Saúde	O trabalho em áreas altamente contaminadas e o uso inadequado de equipamento de proteção individual estiveram associados a 17 sintomas agudos avaliados	NI
Ha et al. ^108^ (2012)	Coreia do Sul	Examinar o estado de exposição e os efeitos agudos à saúde em voluntários limpeza do derramamento de óleo	Observacional sem grupo controle 565 respondentes de uma amostra inicial de 724 3.e	Saúde	Os sintomas físicos estavam associados a durações mais longas de trabalho e níveis significativamente mais altos de metabólitos após limpeza	NI
Pérez-Pereira et al. ^109^ (2012)	Espanha	Estudar o efeito do derrame de petróleo do Prestige em desempenho acadêmico e comportamento de crianças em sala	Transversal 430 indivíduos 4.b	Saúde	O estudo indica que um ano após o derrame, o desastre do Prestige quase não teve consequências nos aspectos avaliados	NI
Zock et al. ^110^ (2012)	Espanha	Avaliar a persistência de sintomas respiratórios 5 anos após o trabalho de limpeza do derramamento do Prestige	Coorte com grupo controle 501 pescadores expostos e 177 não-expostos 3.c	Saúde	Observou-se que a participação em atividades de limpeza de óleo podem resultar em sintomas respiratórios que persistem até 5 anos após a exposição	NI
Cheong et al. ^111^ (2011)	Coreia do Sul	Examinar a relação entre a exposição ao petróleo bruto e os sintomas físicos entre os residentes que participaram do trabalho de limpeza do derramamento de óleo	Observacional sem grupo controle 288 indivíduos 3.e	Saúde	A exposição durante o trabalho de limpeza mostrou associações com os sintomas físico, embora sem anormalidades nos biomarcadores de exposição na urina	NI

BTEX: benzeno, tolueno, etilbenzeno e xilenos; NI: não informado; PUFA: ácidos graxos poli-insaturados; TEPT: transtorno de estresse pós-traumático; THC: hidrocarbonetos totais.

Fonte: elaboração própria.

* Níveis de evidência: 3.c - estudo de coorte com grupo controle; 3.e - estudo observacional sem um grupo controle; 4.b - estudo transversal.

Os estudos identificados foram: Estudos Observacionais sem Grupo Controle (44,44%; nível de evidência 3.e), Estudos de Coorte com Grupo Controle (33,33%; nível de evidência 3.c) e Estudos Transversais (22,22%; nível de evidência 4.b).

Quanto à relação entre objetivos e eixos de investigação dos artigos selecionados, observou-se que a maioria se concentrou no campo da saúde (86,66%), com pouca ênfase no ambiente (4,44%) e nenhuma pesquisa exclusiva sobre aspectos socioeconômicos. Foram identificadas abordagens mistas na revisão: saúde e aspectos socioeconômicos (6,66%); saúde e ambiente (2,22%); e saúde, ambiente e aspectos socioeconômicos (2,22%). Nenhum estudo mencionou a pesquisa de formação preliminar no delineamento metodológico.

Quanto à caracterização dos instrumentos utilizados pelos artigos selecionados (Quadro 3), observou-se que a maioria dos estudos usou questionário próprio aplicado por meio de entrevistas (64,44%), em contraste com o uso isolado de outros instrumentos de avaliação (6,66%). Além disso, foram identificados registros que combinaram diferentes instrumentos (28,88%).

**Quadro 3:** Caracterização dos instrumentos.

ESTUDO (ANO)	INSTRUMENTO DE MEDIDA, AVALIAÇÃO E ACOMPANHAMENTO			
	IDENTIFICAÇÃO	APLICAÇÃO DO INSTRUMENTO PELO ESTUDO	DIMENSÃO ANALISADA	INFORMAÇÕES ADICIONAIS E PADRÕES DE RESPOSTAS DOS INSTRUMENTOS
Denic-Roberts et al. ^71^ (2023)	Questionário próprio para aplicação em método de entrevista	O instrumento considerou a exposição ao derramamento de óleo para estabelecer correlação com uma lista de condições neurológicas crônica autorrelatadas, a partir da CID, incluindo-se enxaqueca, distúrbios de memória e cognição, distúrbios periférios, de sensibilidade, de equilíbrio e marcha	Saúde Aspectos neurológicos	Escala Binária (sempre/nunca): vias de exposição - inalação; contato direto; ingestão; imersão
				Escala de 5 Pontos (nunca/raramente/às vezes/na maioria da vezes/todo o tempo): vias de exposição - inalação; contato direto; ingestão; imersão
Denic-Roberts et al. ^72^ (2022)	Questionário próprio para aplicação em método de entrevista	O instrumento considerou a exposição ao derramamento de óleo para estabelecer correlação com uma lista de condições cardiovasculares autorrelatadas, a partir da CID, incluindo-se hipertensão arterial, aterosclerose coronariana, doenças isquêmicas, doenças de condução, disritimias, embolismo e trombose, e outros sintomas envolvendo o sistema cardiovascular	Saúde Aspectos cardiovasculares	Escala Binária (sempre/nunca): vias de exposição - inalação; contato direto; ingestão; imersão
				Escala de 5 Pontos (nunca/raramente/às vezes/na maioria da vezes/todo o tempo): vias de exposição - inalação; contato direto; ingestão; imersão
				Escala de 3 Pontos (nunca/às vezes/na maioria da vezes): sintomas cardiovasculares agudos - dor no peito; alteração repentina em pulso
Goldman et al. ^73^ (2022)	GSPS	Instrumento utilizado para a coleta de dados referentes às experiências relacionadas ao derramamento de óleo do Deepwater Horizon e à saúde mental da comunidade	Geral Percepções de exposição e impactos	Escala Binária (sim/não): contato com o óleo derramado, em 16 itens relacionados a esta medida de exposição
	GAD-7	Instrumento utilizado para a autoavaliação em casos moderados a graves de ansiedade generalizada. As pontuações, 0 a 21, foram correlacionadas ao dados obtidos pelo GSPS	Saúde Aspectos mentais e emocionais	Escala de 4 Pontos (nenhuma vez (0)/vários dias (1)/mais da metade dos dias (2)/quase todos os dias (3)): 7 perguntas relacionadas à frequência de problemas relacionados à ansiedade
Ferreira et al. ^74^ (2022)	Questionário próprio para aplicação em método de entrevista	Os autores relatam o uso de quatro tipos de questionários para geração de banco de dados específico, no entanto não encontrado em fonte de busca disponibilizada	Multidimensional Aspectos socioeconômicos e de saúde geral	No detalhamento metodológico deste estudo, não há informação suficiente para caracterizar elementos do instrumento e pontos avaliados
Kwok et al. ^75^ (2022)	Questionário próprio para aplicação em método de entrevista	O instrumento considerou a exposição ao derramamento de óleo para estabelecer correlação com medidas de pressão arterial coletadas durante encontros com os indivíduos entrevistados	Saúde Aspectos cardiovasculares	No detalhamento metodológico deste estudo, não há informação suficiente para caracterizar elementos do instrumento e pontos avaliados
Lawrence et al. ^76^ (2022)	Questionário próprio para aplicação em método de entrevista	O instrumento considerou a exposição ao derramamento de óleo para estabelecer correlação com a ocorrência de sintomas respiratórios relacionados à asma, enfisema e bronquite. Para este estudo, também foram realizadas estimativas de exposição associadas por amostras dosimétricas de combustão e de qualidade do ar	Saúde Aspectos respiratórios	Escala de 5 Pontos (nunca/raramente/às vezes/na maioria da vezes/todo o tempo): ocorrência de sibilos como sugestivo de asma
				Escala Binária (sim/não): para casos incidentes de asma, enfisema e bronquite diagnosticados por avaliação médica
				Escala Binária (sim/não): para exposição ao trabalho de contenção do derramamento de óleo
Oghenetega et al. ^77^ (2022)	Questionário próprio para aplicação em método de entrevista	O instrumento considerou a exposição ao derramamento de óleo para estabelecer correlação com os desfechos maternos e resultados neonatais	Saúde Aspectos obstétricos e neonatais	Escala Combinada (sim/não); (não contaminado/levemente contaminado/contaminado); (seguro/ligeiramente contaminado/inseguro e contaminado/muito inseguro/altamente contaminado): para características de exposição à poluição por óleo
				Escala Combinada (sim/não); (nunca/diariamente/uma ou duas vezes na semana/uma vez na semana ou mais/nenhuma resposta/outros) & Padrões de Respostas Livres: para características maternas e estilos de vida - número de gestações; natimorto anterior; morte infantil anterior; tabagismo; consumo de álcool
Rusiecki et al. ^78^ (2022)	Questionário próprio para aplicação em método de entrevista	O instrumento considerou a exposição ao derramamento de óleo para estabelecer correlação com uma lista de condições respiratórias autorrelatadas, a partir da CID, incluindo-se doença pulmonar obstrutiva crônica, rinite, sinusite, bronquite, asma, dispneia, sibilos, tosse e sintomas torácicos	Saúde Aspectos respiratórios	Escala Binária (sempre/nunca): vias de exposição - inalação; contato direto; ingestão; imersão
				Escala de 5 Pontos (nunca/raramente/às vezes/na maioria da vezes/todo o tempo): vias de exposição - inalação; contato direto; ingestão; imersão
Choi et al. ^15^ (2021)	Questionário próprio para aplicação em método de entrevista	O instrumento considerou a exposição ao derramamento de óleo a partir do trabalho de limpeza e a sua duração autorrelatada durante a entrevista para correlacionar aos sintomas psicológicos	Geral Percepções de exposição e impactos	Escala Combinada (sim/não/desconhecido); [Q1 (2 < 12 dias), Q2 (12 < 48 dias), Q3 (48 < 97 dias) e Q4 (97-400 dias)]: para respostas relacionadas ao trabalho e duração, respectivamente
	PDS	A escala utilizada consiste em 17 itens relacionados a sintomas de TEPT, traduzido para o coreano e também validado	Saúde Aspectos mentais e emocionais	Escala de 4 Pontos (nunca (0)/um pouco (1)/muito (2)/sempre (3)): o escore com pontuação acima de 15 foi considerado como risco para TEPT
	CES-D	A escala utilizada consiste em 20 itens relacionados a sintomas depressivos, traduzido para o coreano e também validado	Saúde Aspectos mentais e emocionais	Escala de 4 Pontos (raramente ou nunca (0)/1 ou 2 dias (1)/3 ou 4 dias (2)/sempre (3)): o escore com pontuação acima de 21 foi considerado como risco para depressão
	PWI-SF	O formulário utilizado consiste em 18 itens com perguntas relacionadas ao bem-estar psicossocial, traduzido para o coreano e também validado	Saúde Aspectos mentais e emocionais	Escala de 4 Pontos (nunca (0)/às vezes (1)/com frequência (2)/sempre (3)): o escore com pontuação acima de 27 foi considerado como risco para mal-estar psicossocial
	STAI	O inventário utilizado consiste em 20 itens de avaliação de components subjetivos relacionados à ansiedade, traduzido para o coreano e também validado	Saúde Aspectos mentais e emocionais	Escala de 4 Pontos (nunca (1)/às vezes (2)/com frequência (3)/sempre (4)): o escore com pontuação acima de 52 foi considerado como risco para ansiedade
Eleke et al. ^79^ (2021)	Questionário próprio para aplicação em método de entrevista	O instrumento considerou os efeitos da exposição ao derramamento de óleo sobre as condições gestacionais, perinatais e neonatais a partir das respostas colhidas	Saúde Aspectos obstétricos e neonatais	No detalhamento metodológico deste estudo, não há informação suficiente para caracterizar elementos do instrumento e pontos avaliados
Harville et al. ^80^ (2021)	Questionário próprio para aplicação em método de entrevista	O instrumento considerou a exposição ao derramamento de óleo a partir do trabalho de limpeza e a sua duração autorrelatada durante a entrevista para correlacionar aos impactos socioeconômicos e na saúde gestacional das mulheres	Saúde Aspectos obstétricos e neonatais	Padrões de Respostas Livres: para o envolvimento no trabalho de limpeza e contato com o óleo, exposição direta e efeitos socioeconômicos relacionados à exposição
				Padrões de Respostas Livres: para os desfechos relacionados ao histórico reprodutivo e o resultado final de cada gestação, além do peso ao nascer
Bergstrand & Mayer ^81^ (2020)	CERA (*Gulf Coast Module*) adaptado	A adaptação deste instrumento foi utilizada na abordagem relacionada ao meio ambiente, preocupação ambiental, otimismo da comunidade e variáveis de emprego, além dos efeitos gerais do derramamento de óleo e da percepção de recuperação ao longo do tempo	Multidimensional Percepção de exposição e de impacto ambiental; aspectos socioeconômicos e de saúde geral	Escala de 6 Pontos (discordo completamente (0)/concordo um pouco (1)/concordo de alguma forma (2)/concordo moderadamente (3)/concordo muito (4)/concordo completamente (5)): para avaliação do apoio da comunidade
				Escala de 6 Pontos (nada (0)/um pouco (1)/de alguma forma (2)/moderadamente (3)/ muito (4)/completamente (5)): para percepções de recuperação em quatro cenários - economia, ambiente, comunidade e saúde
				Padrões de Respostas Livres: para interpretações subjetivas de coesão social
				Escala Binária (grandes efeitos (1)/pouco ou nenhum efeito ou não sei (0)): para avaliação das percepções de problemas ambientais
				Escala Binária (lugar melhor/lugar pior): para avaliação do otimismo em relação à comunidade
				Escala Binária (sim/não): para informações relacionadas a raça, estado civil, renda familiar e nível de escolaridade
Bebeteidoh et al. ^82^ (2020)	Questionário próprio para aplicação em método de entrevista	O instrumento considerou o impacto das atividades de refinarias locais de petróleo bruto, na comunidade e no ambiente, percebidos pelos indivíduos	Ambiental Percepções de riscos e impactos	Escala de 6 Pontos (discordo completamente (0)/concordo um pouco (1)/concordo de alguma forma (2)/concordo moderadamente (3)/concordo muito (4)/concordo completamente (5)): para avaliação do impacto ambiental em 6 cenários - terras agrícolas, rendimentos agrícolas, pesca, rendimentos de pesca, poluição da água e resíduos
Oghenetega et al. ^83^ (2020)	Questionário próprio para aplicação em método de entrevista	O instrumento considerou características sociodemográficas e socioeconômicos, além daquelas relacionadas à exposição à poluição por óleo e história obstétrica de gestantes	Saúde Aspectos obstétricos e neonatais	No detalhamento metodológico deste estudo, não há informação suficiente para caracterizar elementos do instrumento e pontos avaliados
Parker et al. ^84^ (2020)	Questionário próprio para aplicação em método de entrevista	O instrumento considerou níveis de preocupação e experiências traumáticas dos indivíduos expostos a impactos contínuos desde o derramamento de óleo	Multidimensional Aspectos socioeconômicos e de saúde geral	Escala de 4 Pontos (nada preocupado (0)/um pouco preocupado (1)/moderadamente preocupado (2)/muito preocupado (3)): para a preocupação persistente em relação aos impactos contínuos
				Padrões de Respostas Livres: para informações acerca da mobilidade dos indivíduos entre os lugares expostos ao derramamento
				Padrões de Respostas Livres: para memórias relacionadas à exposição
	*Trauma History Screen* (adaptado)	A adaptação deste instrumento foi utilizada na abordagem relacionada a eventos de vitimização ocorridos na vida adulta, distribuída em 12 itens para contagem das experiências traumáticas da vida adulta	Saúde Aspectos mentais e emocionais	Escala Binária (desde os 18 anos/no ultimo ano): para avaliação das experiências traumáticas ao longo do tempo
Erickson et al. ^85^ (2019)	Questionário próprio para aplicação em método de entrevista	O instrumento considerou a exposição ao derramamento de óleo para estabelecer correlação com sintomas físicos associados ao calor	Saúde Aspectos gerais	Escala de 5 Pontos (nunca/raramente/às vezes/na maioria da vezes/todo o tempo): vias de exposição - inalação; contato direto; ingestão; imersão
				Escala Binária (sempre/nunca): para o uso de equipamentos de proteção individual
				Padrões de Respostas Livres: para informações acerca de sintomas relacionados a calor
Kaufman et al. ^86^ (2019)	Questionário próprio para aplicação em método de entrevista	O instrumento considerou a exposição ao derramamento de óleo para estabelecer correlação com sintomas de depressão, além de fatores relacionados a suporte emocional, cargas e participações nos esforços de limpeza do derramamento	Saúde Aspectos mentais e emocionais	Escala Binária (sim/não): para contato com o óleo derramado e participação nos esforços de limpeza
				Escala de 5 Pontos (nunca/raramente/às vezes/na maioria da vezes/todo o tempo): para avaliação do suporte emocional em 5 contextos durante os esforços
	PHQ-8	O instrumento validado foi utilizado para a avaliação autorreferida da frequência de sintomas de depressão nos indivíduos entrevistados	Saúde Aspectos mentais e emocionais	Escala de 4 Pontos (nenhuma vez (0)/vários dias (1)/mais da metade dos dias (2)/quase todos os dias (3)): o escore com pontuação acima de 10 foi considerado como risco para depressão
Krishnamurthy et al. ^87^ (2019)	Questionário próprio para aplicação em método de entrevista	O instrumento considerou a exposição ao derramamento de óleo e às substâncias químicas relacionadas aos esforços de limpeza para estabelecer correlação com sintomas neurológicos agudos	Saúde Aspectos neurológicos	Escala de 5 pontos (nunca/raramente/às vezes/na maioria da vezes/todo o tempo): para a frequência de exposição ao óleo bruto/água oleosa, além dos dispersantes de óleo
				Escala de 3 Pontos (nunca/às vezes/na maioria das vezes): para os sintomas neurológicos autorrelatados
				Padrões de Respostas Livres: para dados sociodemográficos e de exposição ao óleo e dispersantes
Nugent et al. ^88^ (2019)	Questionário próprio para aplicação em método de entrevista	O instrumento considerou a exposição ao derramamento de óleo para estabelecer correlação com os sintomas e a ocorrência de TEPT	Saúde Aspectos mentais e emocionais	Padrões de Respostas Livres: para dados sociodemográficos, de exposição e de percepção do estado de saúde física
				Escala Binária (sim/não): para a capacidade de sentir o cheiro do óleo, perda de renda doméstica e impacto na situação financeira
				Escala de 5 Pontos (nenhuma influência/uma influência positiva/uma influência/uma influência negativa/uma influência muito negativa): para avaliação específica da situação financeira
	*Life Events Checklist*	Instrumento com 20 itens para avaliação de experiências potencialmente traumáticas ao longo da vida	Saúde Aspectos mentais e emocionais	Escala de 5 Pontos (aconteceu comigo/testemunhei isso/aprendi sobre isso/não tenho certeza/não se aplica): para avaliação específica da situação financeira
Quist et al. ^19^ (2019)	Questionário próprio para aplicação em método de entrevista	O instrumento considerou a exposição ao derramamento de óleo e às substâncias químicas relacionadas aos esforços de limpeza para estabelecer correlação com a função neurocomportamental	Saúde Aspectos mentais e emocionais	No detalhamento metodológico deste estudo, não há informação suficiente para caracterizar elementos do instrumento e pontos avaliados
	BARS	O instrumento foi utilizado para a avaliação neurocomportamental, incluindo uma série de testes com instruções verbais simples e gráficos de respostas. Cada teste apresenta uma ou mais medidas de resultado para avaliar fatores como latência de resposta, erro e respostas corretas	Saúde Aspectos mentais e emocionais	Teste de Desempenho Contínuo: para avaliar atenção visual sustentada e a memória de curto prazo
				Teste de Extensão de Dígitos: para avaliar a atenção e a memória
				Teste de Correspondência com Amostra: para avaliar memória visual
				Teste Dígito-Símbolo: para avaliar função executiva e codificação
				Teste de Tempo de Reação Simples: para avaliar velocidade de resposta
				Teste de Batida de Dedos: para avaliar velocidade de resposta e coordenação
				Teste de Proporção Progressiva: para avaliar motivação relacionada ao esforço
Rung et al. ^89^ (2019)	Questionário próprio para aplicação em método de entrevista	O instrumento considerou a exposição ao derramamento de óleo para estabelecer correlação com os impactos físicos e socioeconômicos	Geral Percepções de exposição e impactos	No detalhamento metodológico deste estudo, não há informação suficiente para caracterizar elementos do instrumento e pontos avaliados, embora os autores citem o agrupamento de itens em “exposição econômica” e “exposição física” para registro da frequência dos relatos
	CES-D	Instrumento projetado para estudos epidemiológicos, que permite a avaliação de sintomas depressivos e variações de humor. Neste estudo, a escala foi utilizada como um medidor contínuo dos sintomas	Saúde Aspectos mentais e emocionais	Escala de 4 Pontos (raramente ou nunca (0)/um ou dois dias (1)/três ou quatro dias (2)/sempre (3)): o escore com pontuação acima ou igual a 16 foi considerado como risco para depressão
	K6	Instrumento de avaliação do sofrimento psicológicos não específico utilizado no rastreio de transtornos de humor e de ansiedade. Neste estudo, a escala foi utilizada como um medidor contínuo dos sintomas	Saúde Aspectos mentais e emocionais	Escala de 5 Pontos (nunca (1)/raramente (2)/às vezes (3)/na maioria da vezes (4)/todo o tempo (5)): o escore com pontuação acima ou igual a 13 foi considerado como provável sofrimento mental grave
Strelitz et al. ^90^ (2019)	Questionário próprio para aplicação em método de entrevista	O instrumento considerou a exposição ao derramamento de óleo e o tempo de duração no trabalho de limpeza para estabelecer correlação com a ocorrência de um primeiro evento de doença cardíaca autorreferida ou fatal, neste último, avaliados pelo Índice Nacional de Óbitos	Saúde Aspectos cardiovasculares	Padrões de Respostas Livres: para dados sociodemográficos, de duração da exposição, duração no trabalho de limpeza e proximidade residencial, além do diagnóstico de evento cardíaco
Strelitz et al. ^91^ (2019)	Questionário próprio para aplicação em método de entrevista	O instrumento considerou a exposição ao derramamento de óleo a partir de atividades autorrelatadas, associada a marcadores gerais de hidrocarbonetos, para realizar correlação com a ocorrência de eventos cardíacos	Saúde Aspectos cardiovasculares	Padrões de Respostas Livres: para dados relacionados às atividades e aos padrões de trabalho que envolvem exposição, além dos desfechos de interesse para um primeiro incidente cardíaco e fatores demográficos e de estilos de vida
Werder et al. ^92^ (2019)	Questionário próprio para aplicação em método de entrevista	O instrumento considerou a ocorrência de sintomas neurológicos dos indivíduos entrevistados para realizar correlação com a exposição às substâncias químicas relacionadas ao trabalho de limpeza do óleo derramado	Saúde Aspectos neurológicos	Escala de 5 Pontos (nunca/raramente/às vezes/na maioria da vezes/todo o tempo): para o autorrelato da frequência de sintomas neurológicos
				Padrões de Respostas Livres: para informações sociodemográficas e relacionadas à exposição às substâncias químicos
Alexander et al. ^93^ (2018)	Questionário próprio para aplicação em método de entrevista	O instrumento considerou a ocorrência de sintomas respiratórios agudos dos indivíduos entrevistados para realizar correlação com a exposição ao derramamento de óleo e às substâncias químicas relacionadas ao trabalho de limpeza	Saúde Aspectos respiratórios	Escala de 5 Pontos (nunca/raramente/às vezes/na maioria da vezes/todo o tempo): para a frequência de exposição ao petróleo bruto/água oleosa e dispersantes em quatro contextos - inalação, contato direto com a pele, ingestão e submersão
				Escala de 3 Pontos (nunca/às vezes/na maioria das vezes): para os sintomas respiratórios agudos autorrelatados
Harville et al. ^94^ (2018)	Questionário próprio para aplicação em método de entrevista	O instrumento considerou a ocorrência de abortos espontâneos e infertilidade entre as mulheres entrevistadas para realizar correlação com a exposição ao derramamento de óleo	Saúde Aspectos obstétricos e neonatais	Escala Combinada (algum/nenhum); (nenhum/algum/muito): para as consequências financeiras/de renda, contato direto com o óleo, trauma relacionado ao derramamento de óleo e perda de uso da costa
				Escala Binária (sim/não): para a ocorrência de mudanças comportamentais relacionadas ao derramamento
				Padrões de Respostas Livres: para informações relacionadas ao histórico reprodutivo das mulheres entrevistadas, além das suas mudanças comportamentais
Rusiecki et al. ^95^ (2018)	Questionário próprio para aplicação em método de entrevista	O instrumento considerou a ocorrência de sintomas respiratórios/neurológicos/geniturinários/cardiovasculares agudos dos indivíduos entrevistados para realizar correlação com a exposição ao derramamento de óleo e às substâncias químicas relacionadas ao trabalho de limpeza	Saúde Aspectos gerais	Escala Binária (sempre/nunca): para a relação autorrelatada entre exposição e efeitos na saúde
				Escala de 5 Pontos (nunca/raramente/às vezes/na maioria da vezes/todo o tempo): para a frequência de exposição ao petróleo bruto/água oleosa e dispersantes, além de gases de escapamento
				Escala de 3 Pontos (nunca/às vezes/na maioria das vezes): para os sintomas respiratórios/neurológicos/geniturinários/cardiovasculares agudos autorrelatados
				Padrões de Respostas Livres: para informações relacionadas ao trabalho e à exposição ao derramamento de óleo, além do uso de equipamentos de proteção individual, sintomas agudos experimentados e fatores de estilos de vida
Strelitz et al. ^96^ (2018)	Questionário próprio para aplicação em método de entrevista	O instrumento considerou a exposição ao derramamento de óleo a partir de atividades autorrelatadas, associada à proximidade residencial, para realizar correlação com a ocorrência de infarto do miocárdio não fatal	Saúde Aspectos cardiovasculares	Padrões de Respostas Livres: para dados relacionados às atividades e aos padrões de trabalho que envolvem exposição, além dos desfechos de interesse para infarto de miocárdio não fatal e fatores demográficos e de estilos de vida
				Escala Binária (sim/não): para a participação no trabalho de limpeza, trabalho com queima de petróleo bruto e interrupção por motivos de exposição térmica
				Escala Binária (direta/indireta): para a proximidade residencial do local exposto ao derramamento de óleo
				Escala de 4 Pontos (1-30 dias/31-90 dias/91-180 dias/> 180 dias): para a duração do trabalho de limpeza
Croisant et al. ^17^ (2017)	Questionário próprio para aplicação em método de entrevista	O instrumento considerou a percepção geral de saúde e fatores sociais associados para realizar correlação com a exposição ao derramamento de óleo e a outros instrumentos de medida	Geral Percepções de exposição e impactos	Escala de 5 Pontos (muito ruim/ruim/regular/bom/muito bom): para itens relacionados à percepção geral da saúde
				Escala Binária (sim/não): para diagnóstico de hipertensão, diabetes, cardiopatias, cerebropatias ou cancer, além de acesso a serviços de saúde e impactos econômicos e na condução de vida
				Escala de 5 Pontos (nunca/raramente/às vezes/na maioria da vezes/todo o tempo): para itens relacionados ao apoio social
	GAD-7	Instrumento utilizado para a autoavaliação em casos de ansiedade generalizada. As pontuações para os 7 itens foram somadas (intervalo de 0 a 21) e interpretadas como ansiedade mínima (0 a 4), ansiedade leve (5 a 9), ansiedade moderada (10 a 14), e ansiedade severa (15 a 21)	Saúde Aspectos mentais e emocionais	Escala de 4 Pontos (0-1 dia (0)/2-6 dias (1)/7-11 dias (2)/12-14 dias (3)): 7 perguntas relacionadas à frequência de problemas relacionados à ansiedade
	PHQ-8	Instrumento utilizado para a avaliação autorreferida da frequência de sintomas de depressão nos indivíduos entrevistados. As pontuações para sintomas depressivos foram interpretadas como nenhum (0-4), leve (5-9), moderada (10-14), moderadamente grave (15-19) e grave (20-24)	Saúde Aspectos mentais e emocionais	Escala de 4 Pontos (0-1 dia (0)/2-6 dias (1)/7-11 dias (2)/12-14 dias (3)): 7 perguntas relacionadas à frequência de problemas relacionados a sintomas de depressão
	PC-PTSD	Instrumento de 4 itens utilizado para a avaliação de TEPT. A presença foi interpretada como uma pontuação de 3 ou mais	Saúde Aspectos mentais e emocionais	Escala Binária (sim/não): para a sintomas do transtorno observados durante os últimos 30 dias
	*Self-Mastery Scale*	Instrumento de quatro itens utilizado para a avaliação de resiliência e capacidade de enfrentamento. Pontuações mais altas indicando níveis mais altos de autodomínio	Saúde Aspectos mentais e emocionais	Escala de 5 Pontos (discordo completamente (1)/concordo um pouco (2)/concordo de alguma forma (3)/ concordo moderadamente (4)/ concordo completamente (5)): para itens relacionados ao autodomínio
Harville et al. ^18^ (2017)	Questionário próprio para aplicação em método de entrevista	O instrumento considerou a ocorrência de efeitos físicos e socioeconômicos, além de complicações na gravidez, entre as mulheres entrevistadas para realizar correlação com a exposição ao derramamento de óleo	Multidimensional Aspectos socioeconômicos e de saúde obstétrica	Escala Combinada (algum/nenhum); (nenhum/algum/muito): para as consequências financeiras/de renda, contato direto com o óleo, trauma relacionado ao derramamento de óleo e perda de uso da costa
				Padrões de Respostas Livres: para informações relacionadas ao envolvimento no trabalho de limpeza e exposição direta ao derramamento de óleo
				Padrões de Respostas Livres: para informações relacionadas ao histórico reprodutivo das mulheres entrevistadas
Kwok et al. ^97^ (2017)	Questionário próprio para aplicação em método de entrevista	O instrumento considerou a ocorrência de efeitos na saúde mental entre os indivíduos entrevistados para realizar correlação com a exposição ao derramamento de óleo	Saúde Aspectos mentais e emocionais	Padrões de Respostas Livres: para informações relacionadas ao envolvimento no trabalho de limpeza e exposição direta ao derramamento de óleo
	PHQ-8	Instrumento utilizado para a avaliação autorreferida da frequência de sintomas de depressão nos indivíduos entrevistados. Os escores de 10 ou mais sugerindo uma provável indicação de depressão moderada a grave	Saúde Aspectos mentais e emocionais	Escala de 4 Pontos (0-1 dia (0)/2-6 dias (1)/7-11 dias (2)/12-14 dias (3)): 7 perguntas relacionadas à frequência de problemas relacionados a sintomas de depressão
	PC-PTSD	Instrumento de 4 itens utilizado para a avaliação de TEPT. A presença foi interpretada como uma pontuação de 3 ou mais	Saúde Aspectos mentais e emocionais	Escala Binária (sim/não): para a sintomas do transtorno observados durante os últimos 30 dias
McGowan et al. ^98^ (2017)	Questionário próprio para aplicação em método de entrevista	O instrumento considerou a ocorrência de sintomas respiratórios, oculares e dérmicos entre os indivíduos entrevistados para realizar correlação com a exposição ao derramamento de óleo	Saúde Aspectos gerais	Escala Binária (sempre/nunca): para sintomas respiratórios, irritação ocular e dérmica, e exposição pelo trabalho e aos dispersantes usados na limpeza
				Escala de 5 Pontos (nunca/raramente/às vezes/na maioria da vezes/todo o tempo): para a frequência dos sintomas apresentados
Zilversmit et al. ^99^ (2017)	Questionário próprio para aplicação em método de entrevista	O instrumento considerou a frequência de consumo de alimentos marinhos entre os indivíduos entrevistados para realizar correlação com os níveis de biomarcadores no sangue após a exposição	Saúde Aspectos gerais	Escala de 8 Pontos (nunca/menos de 1 vez por mês/1 vez por mês/2-3 vezes por mês/1-2 vezes por semana/3-4 vezes por semana/5-6 vezes por semana/1 ou mais vezes por dia): para a frequência de consumo de alimentos marinhos
Nriagu et al. ^100^ (2016)	GHQ adaptado	A adaptação deste instrumento foi utilizada na abordagem relacionada à limitação da capacidade funcional, distribuída em 9 itens para avaliar a o nível de limitação para uma atividades diária típica, como ir ao mercado, pescar e cultivar. A pontuação total da escala (variando de 4 a 36) foi classificada em 4-9 (baixo), 10-20 (médio) e 21-36 (alta)	Saúde Aspectos gerais	Escala de 4 Pontos (menos do que o normal (1)/não mais do que o habitual (2)/um pouco mais do que o normal (3)/muito mais do que o normal (4)): 7 perguntas relacionadas à frequência de problemas relacionados a sintomas de depressão
	EEQs	4 questões de exposição ambiental para avaliar a perspectiva de exposição ambiental em termos de distância residencial e frequência de contato direto com a poluição por óleo	Ambiental Percepções de riscos e impactos	Q1 e Q2 (< 50m, 50-100m, 100-500m e > 500m): para distância residencial até o local de poluição visível e de instalações de queima de gás, respectivamente; Q3 Escala de 4 Pontos (nunca (1)/1-5 vezes (2)/5-10 vezes (3)/mais de 10 vezes (4)): para frequência de exposição à poluição; Q4 Escala de 4 Pontos (não contaminada (1)/um pouco contaminada (2)/muito contaminada (3)/altamente contaminada (4)): para nível de poluição da água potável
	Questionário de autorrelato de eventos de risco	A adaptação desta escala foi utilizada na abordagem relacionada ao risco ambiental percebido. A faixa de pontuação total para a escala (4-28) foi categorizada em 4-10 (baixo), 11-19 (médio), 20-28 (alto)	Ambiental Percepções de riscos e impactos	Escala de 4 Pontos (nada preocupado (1)/um pouco preocupado (2)/moderadamente preocupado (3)/muito preocupado (4)): 7 perguntas relacionadas ao risco ambiental percebido
	ERT	Escala padronizada para a avaliação da tolerância ao risco ambiental, a partir de 11 declarações relacionadas à poluição por óleo. A escala foi subcategorizada em 0-5 (tolerância mínima), 6-15 (baixa), 16-25 (média) e 26-44 (alta tolerância). A pontuação reversa nas escalas dá uma indicação de intolerância ao risco ambiental	Ambiental Percepções de riscos e impactos	Escala de 4 Pontos (nada preocupado (1)/um pouco preocupado(2)/moderadamente preocupado (3)/muito preocupado(4)): 7 perguntas relacionadas ao risco ambiental percebido
	EHA	Escala para avaliação de aborrecimento associado à percepção de risco ambiental, a partir de 12 perguntas gerais de eventos ambientais adversos. A pontuação total da escala foi categorizada em 4-10 (mínima), 11-20 (baixa), 21-30 (média) e 31-48 (alta) para a análise de dados	Ambiental Percepções de riscos e impactos	Escala de 4 Pontos (nada preocupado (1)/um pouco preocupado(2)/moderadamente preocupado (3)/muito preocupado(4)): para perguntas relacionadas a aspectos de aborrecimento após percepção de risco ambiental
	PSW	Inventário para a avaliação de intensidade e excesso de preocupação sobre um conteúdo específico, neste caso, a poluição por petróleo. A pontuação total potencial de 52 foi subdividida em 4-10 (mínimo), 11-20 (baixo), 21-35 (médio) e 36-52 (alto)	Saúde Aspectos mentais e emocionais	Escala de 4 Pontos (nada preocupado (0)/um pouco preocupado (1)/moderadamente preocupado (2)/muito preocupado (3)): para perguntas relacionadas à preocupação patológica associada à poluição por petróleo
	HSI	Escala utilizada para a avaliação da carga sintomática de doenças nas famílias dos participantes. As respostas foram somadas para cada indivíduo, resultando em pontuação total que variou de 0 a 44. A pontuação foi subdividida em 0-5 (mínimo), 6-15 (baixo), 16-25 (médio) e 26-44 (alto)	Saúde Aspectos mentais e emocionais	Escala Binária (sim/não): para sintomas gerais relacionados após exposição
Peres et al. ^101^ (2016)	Questionário próprio para aplicação em método de entrevista	O instrumento considerou a ocorrência de sintomas gerais entre os indivíduos entrevistados para realizar correlação com a exposição ao derramamento de óleo	Saúde Aspectos gerais	Escala Combinada (sim/não); (mais afetado/igual/menos afetado)/(muito negativo/algo negativo/algo positivo/muito positivo/sSem influência); (sem força/pouco forte/moderadamente forte/muito forte/completamente forte); (nunca/um pouco/alguma vez/na maior parte do tempo/sempre): para perguntas relacionadas à exposição ao derramamento de óleo
				Escala de 5 Pontos (nunca/raramente/às vezes/na maioria da vezes/todo o tempo): para a frequência dos sintomas apresentados
Rung et al. ^102^ (2016)	Questionário próprio para aplicação em método de entrevista	O instrumento considerou os efeitos da exposição ao derramamento de óleo para realizar correlação com os desfechos de saúde mental	Geral Percepções de exposição e impactos	Escala Binária (sim/não): para consequências econômicas relacionadas ao derramamento de petróleo e seus níveis de exposição
				Escala Binária (influência negativa/influência positiva): para a influência do derramamento na situação financeira familiar
	CES-D	A escala utilizada consiste em 20 itens relacionados a sintomas depressivos.	Saúde Aspectos mentais e emocionais	Escala de 4 Pontos (raramente ou nunca (0)/1 ou 2 dias (1)/3 ou 4 dias (2)/sempre (3)): a pontuação de corte para sintomas depressivos foi 16
	K6	Instrumento de avaliação do sofrimento psicológicos não específico utilizado no rastreio de transtornos de humor e de ansiedade	Saúde Aspectos mentais e emocionais	Escala de 5 Pontos (nunca (1)/raramente (2)/às vezes (3)/na maioria da vezes (4)/todo o tempo (5)): o escore com pontuação acima ou igual a 13 foi considerado como provável sofrimento mental grave
Simon-Friedt et al. ^103^ (2016)	Questionário próprio para aplicação em método de entrevista	O instrumento considerou riscos específicos percebidos dentro de comunidades após a exposição ao derramamento de petróleo	Ambiental Percepções de riscos e impactos	No detalhamento metodológico deste estudo, não há informação suficiente para caracterizar elementos do instrumento e pontos avaliados
Ha et al. ^104^ (2013)	CDI	Versão coreana do inventário para medição de sintomas de depressão de crianças, composto por 27 questões pontuadas de 0 a 2	Saúde Aspectos mentais e emocionais	Escala de 3 Pontos (um pouco (0)/moderadamente (1)/muito (3)): a pontuação de corte para sintomas de depressão sendo maior ou igual a 22
	SAIC	Versão coreana do inventário para medição de sintomas de ansiedade de crianças, composto por 20 questões pontuadas de 1 a 3	Saúde Aspectos mentais e emocionais	Escala de 3 Pontos (às vezes (1)/frequentemente (2)/sempre (3)): a pontuação de corte para sintomas de ansiedade sendo maior ou igual a 41
Jung et al. ^105^ (2013)	ISAAC modificado	Versão coreana do questionário utilizado para a avaliação de características relacionadas à asma, que foi correlacionado a outras informações associadas a quadro alérgico e teste de função pulmonar	Saúde Aspectos respiratórios	Escala Binária (sim/não): para informações relacionadas ao diagnóstico prévio de asma e a ocorrência de sibilos nos últimos 12 meses
Buttke et al. ^106^ (2012)	CASPER	Instrumento utilizado para realizar a coleta de informações domiciliares sobre as necessidades de uma comunidade afetada após um desastre, envolvendo dados relacionados à saúde física e mental	Saúde Aspectos mentais e emocionais; aspectos gerais	As perguntas foram adaptadas a partir de outras fontes como o BRFSS do CDC dos Estados Unidos, o PHQ-2 (sintomas depressivos) e o GAD-2 (sintomas de ansiedade)
Gwack et al. ^107^ (2012)	Questionário próprio para aplicação em método de entrevista	O instrumento considerou a ocorrência de sintomas agudos neurológicos, respiratórios, dermatológicos, oftalmológicos e gerais entre os indivíduos entrevistados para realizar correlação com a exposição ao derramamento de óleo	Saúde Aspectos gerais	No detalhamento metodológico deste estudo, não há informação suficiente para caracterizar elementos do instrumento e pontos avaliados
Ha et al. ^108^ (2012)	Questionário próprio para aplicação em método de entrevista	O instrumento considerou a ocorrência de sintomas físicos gerais entre os indivíduos entrevistados para realizar correlação com a exposição ao derramamento de óleo e aos níveis de metabólitos urinários associados a químicos utilizados no trabalho de limpeza	Saúde Aspectos gerais	Escala Combinada (1 dia/mais de 1 dia); (diretamente/indiretamente/outros); (nada/um pouco/muito/profundo): para perguntas relacionadas à exposição ao derramamento de óleo
				Escala Binária (sim/não): para a sintomas físicos diversos autorrelatados pelos entrevistados
				Padrões de Respostas Livres: para informações relacionadas questões sociodemográficas, hábitos de vida, história pregressa e uso de equipamentos de proteção individual
Pérez-Pereira et al. ^109^ (2012)	CBI	Inventário utilizado para a avaliação de comportamento de crianças e pré-adolescentes em idade escolar em sala de aula	Saúde Aspectos mentais e emocionais	Composto por 5 subescalas: comportamento inteligente (inteligência verbal, criatividade e curiosidade); extroversão/introversão, consideração/hostilidade, independência/dependência e concentração/distração. No detalhamento metodológico deste estudo, não há informação suficiente para caracterizar elementos do instrumento e pontos avaliados
	Questionário adaptado de Palinkas et al. ^112^	Adaptação utilizada para a avaliação do grau de exposição ao desastre como um índice de risco medido pela proximidade de moradia em relação ao local do evento	Geral Percepções de exposição e impactos	No detalhamento metodológico deste estudo, não há informação suficiente para caracterizar elementos do instrumento e pontos avaliados, embora os autores citem a validação por escala de 6 pontos
	FACES-II	Escala utilizada para a avaliação de dados associados a questões familiares, compreendendo 16 itens sobre coesão e 14 itens de adaptabilidade	Saúde Aspectos mentais e emocionais	No detalhamento metodológico deste estudo, não há informação suficiente para caracterizar elementos do instrumento e pontos avaliados
	CSCY	Escala utilizada para a avaliação de proteção/vulnerabilidade pessoal e estratégias de enfrentamento	Saúde Aspectos mentais e emocionais	Composto por 4 subescalas: busca de assistência; resolução de problemas; estratégias cognitivas evasivas; estratégias comportamentais evasivas. No detalhamento metodológico deste estudo, não há informação suficiente para caracterizar elementos do instrumento e pontos avaliados
Zock et al. ^110^ (2012)	Questionário próprio para aplicação em método de entrevista	O instrumento considerou a persistência de sintomas respiratórios entre os indivíduos entrevistados para realizar correlação com a exposição ao derramamento de óleo	Saúde Aspectos respiratórios	Escala Binária (sim/não): para a sintomas respiratórios e uso de medicamentos inalatórios e orais nos últimos 12 meses
				Escala de 3 Pontos (nunca/anterior/atual): para tabagismo
				Padrões de Respostas Livres: para informações relacionadas ao envolvimento no trabalho de limpeza e exposição direta ao derramamento de óleo
Cheong et al. ^111^ (2011)	Questionário próprio para aplicação em método de entrevista	O instrumento considerou a ocorrência de sintomas físicos entre os indivíduos entrevistados para realizar correlação com a exposição ao derramamento de óleo e taxas de metabólitos urinários relacionados os químicos	Saúde Aspectos gerais	Escala de 4 Pontos (nunca/pouco/muito/profundo): para a frequência dos sintomas físicos subjetivos, características gerais e história pregressa
				Escala de 4 Pontos (menos de 10 dias/10 a menos de 13 dias/13 a menos de 20 dias/20 ou mais dias): para a frequência de exposição ao petróleo bruto

BARS: *Behavioral Assessment and Research System*; BRFSS: *Behavioral Risk Factor Surveillance System*; CASPER: *Community Assessment for Public Health Emergency Response*; CBI: *Classroom Behavior Inventory*; CDC: Centro de Controle e Prevenção de Doenças; CDI: *Children’s Depression Inventory*; CERA: *Community and Environment in Rural America*; CES-D: *20-item Center for Epidemiological Studies Depression Scale*; CID: Classificação Internacional de Doenças; CSCY: *Coping Scale for Children and Youth*; EEQs: *Environmental Exposure Questions*; EHA: *Environmental Hazard Annoyance*; ERT: *Environmental Risk Tolerance*; FACES: *Family Adaptability and Cohesion Evaluation Scales*; GAD-2: *2-item Generalized Anxiety Disorder*; GAD-7: *7-item Generalized Anxiety Disorder*; GHQ: *General Health Questionnaire*; GSPS: *Gulf States Population Survey*; HSI: *Health Symptoms Inventory*; ISAAC: *International Study of Asthma and Allergies in Childhood Questionnaire*; K6: *Kessler-6*; PC-PTSD: *Primary Care PTSD Screen*; PDS: *Posttraumatic Diagnostic Scale*; PHQ-2: *2-item Patient Health Questionnaire*; PHQ-8: *8-item Patient Health Questionnaire*; PSW: *The Penn State Worry Questionnaire*; PWI-SF: *Psychosocial Well-Being Index-Short Form*; SAIC: *State-Trait Anxiety Inventory for Children*; STAI: *State-Trait Anxiety Inventory*; TEPT: transtorno de estresse pós-traumático.

Fonte: elaboração própria.

Ao total, foram observados 75 registros de uso de instrumentos. Além dos questionários próprios, foram identificados 29 instrumentos distintos para avaliar os impactos dos derramamentos de petróleo. Entre eles, o *20-item Center for Epidemiological Studies Depression Scale* (CES-D) e o *8-item Patient Health Questionnaire* (PHQ-8) foram os mais utilizados, cada um em três estudos. Seguiram-se o *7-item Generalized Anxiety Disorder* (GAD-7), o *Kessler-6* (K6) e o *Primary Care PTSD* (PC-PTSD) screen, cada um em dois estudos. Apenas quatro instrumentos focaram em aspectos ambientais e percepções dos respondentes: *Community and Environment in Rural America Gulf Coast Module*, *Environmental Exposure Questions*, *Risky Events Self-Report Questionnaire* e *Environmental Risk Tolerance*. Outros instrumentos podem ser consultados no Quadro 3.

Nove artigos não forneceram informações suficientes sobre os instrumentos utilizados, limitando a análise detalhada. Observou-se o uso de escalas binárias, escalas do tipo Likert, escalas combinadas e respostas livres na estruturação dos instrumentos (Quadro 3).

Dentre as dimensões analisadas pelos instrumentos (Quadro 3), observou-se o predomínio do foco em aspectos mentais e emocionais na dimensão saúde (41,33%), seguido por aspectos gerais da saúde física (13,33%), e aspectos cardiovasculares, respiratórios e obstétricos/neonatais (6,66% cada). Aspectos neurológicos foram abordados em 2,66% dos estudos.

Na dimensão geral, foram analisadas percepções de exposição e impacto (8%), enquanto na dimensão ambiental, foram avaliadas percepções de riscos ambientais e impactos (8%). Também foram identificadas variáveis multidimensionais para avaliação de aspectos socioeconômicos e saúde geral (2,66%), e aspectos socioeconômicos com saúde obstétrica, percepção de exposição e impacto ambiental (1,33% cada).

Considerando os Quadros 2 e 3 2os objetivos dos artigos, a exposição ao petróleo/óleo bruto e as respostas de contenção estão associadas aos seguintes achados:

(a) Na saúde: aumento de distúrbios neurológicos e comportamentais, como transtorno de ansiedade, depressão e transtorno de estresse pós-traumático (TEPT); distúrbios cardiovasculares e respiratórios, como hipertensão, infarto, asma e doenças pulmonares; e eventos obstétricos e neonatais adversos, como abortos e natimortos.

(b) No ambiente: percepções de aumento do risco associado à exposição e impactos na vida marinha, levando à redução do consumo de frutos do mar.

(c) No contexto socioeconômico: impactos negativos no turismo e na pesca, perda de subsistência para trabalhadores locais e alterações nas rotas de cultivo, destacando a importância da coesão social na recuperação dos danos.

## Discussão

Os estudos identificados mostram uma variedade de instrumentos utilizados em desastres de derramamento de petróleo, embora a maioria apresente modelos próprios dos grupos de pesquisa e métodos de coleta de dados baseados em escalas para investigar os impactos. Esses impactos afetam tanto o ambiente quanto os seres humanos, especialmente a população costeira e os territórios próximos, gerando grande vulnerabilidade ^33^. Isso ressalta a importância de compreender as mudanças nas dinâmicas socioeconômicas, na ocupação dos espaços e na determinação do processo saúde-doença nessas áreas ^34^.

Ao comparar o gerenciamento de desastres relacionados a derramamentos de petróleo com outros desastres naturais, tecnológicos e químicos, identificam-se tanto potenciais quanto limitações. Entre os potenciais, destacam-se a melhora na coordenação e resposta rápida, o uso de tecnologias avançadas e a capacitação eficaz das equipes. Estratégias de comunicação claras e a colaboração entre instituições também otimizam recursos e promovem uma resposta integrada. No entanto, limitações como avaliação e gerenciamento inadequados podem causar atrasos no acesso às áreas afetadas, aumento do número de vítimas, sofrimento psicológico, perda de coordenação nas ações de socorro, destruição cultural, maior vulnerabilidade e migração em busca de recursos ^35^.

Portanto, é crucial aprimorar continuamente as metodologias de avaliação para lidar com a diversidade de cenários e a complexidade dos impactos sobre as comunidades afetadas. A análise dos instrumentos utilizados, especialmente em desastres provocados por derramamentos de petróleo, revela a importância de ferramentas como o CES-D, PHQ-8, GAD-7, K6 e PC-PTSD. Esses instrumentos são eficazes para a triagem e o monitoramento de condições psicológicas pós-desastre, sendo padronizados e de fácil aplicação para identificar sintomas de depressão, ansiedade e TEPT. Contudo, apesar de sua utilidade, esses instrumentos podem não oferecer um diagnóstico completo e não captar a complexidade das experiências vividas pelas populações afetadas, bem como os fatores sociais, econômicos e culturais que contribuem para o desenvolvimento de transtornos mentais ^36^ ^,^ ^37^ ^,^ ^38^ ^,^ ^39^ ^,^ ^40^ ^,^ ^41^.

No contexto da saúde, a exposição ao petróleo está associada a sintomas físicos e mentais, genotóxicos e endócrinos, de diferentes gravidades em vários sistemas orgânicos. Além disso, tanto a intoxicação aguda quanto a crônica aumentam a vulnerabilidade da saúde mental, especialmente em indivíduos afetados pela destruição dos territórios e perda dos mecanismos de sobrevivência ^42^. Destaca-se o predomínio de artigos focados na saúde mental, justificado pela preocupação com o aumento de desordens na vida, no trabalho, na família e na vida social dos sobreviventes, bem como os impactos nas atividades de subsistência e a perda econômica ^43^.

Os instrumentos também revelaram percepções sobre impactos e riscos ambientais, além dos efeitos negativos nas condições socioeconômicas das populações expostas, semelhantes aos encontrados em estudos sobre a ocupação de territórios por indústrias poluentes, e a insustentabilidade ambiental dessas atividades ^44^.

É importante ressaltar que os artigos selecionados para esta revisão não se debruçam sobre os aspectos íntimos das injustiças ambientais e vulnerabilização socioeconômica causados pela exposição ao petróleo. Isso contribui para uma produção de conhecimento enviesado que oculta contextos de risco e desfavorece grupos humanos vulneráveis nesse modelo de concepção da avaliação dos impactos ^10^.

A falta de estudos sobre as percepções e respostas das comunidades afetadas por derramamentos de petróleo pode levar a intervenções inadequadas e déficits em políticas, agravando disparidades e injustiças, e intensificando o impacto do racismo ambiental. Esta revisão destaca a necessidade de uma abordagem interdisciplinar e sistêmica, com políticas públicas integradas, para entender a relação entre saúde, ambiente e aspectos sociais e econômicos, e abordar os efeitos adversos sobre condições de vida e ocupação de territórios.

Ademais, sugere-se fortalecer a articulação entre os diferentes níveis de governo, com destaque para o nível municipal, em que se implementa e executa diretamente a política formulada, fortalecendo a participação social e permitindo que as comunidades locais colaborem na construção de soluções para as suas realidades específicas, nesse caso, o enfrentamento dos desastres ^45^ ^,^ ^46^ ^,^ ^47^.

Diante dos desafios na gestão de desastres, é importante adotar medidas como padronização de protocolos de resposta rápida, sistemas de monitoramento ambiental e mecanismos de fiscalização robustos, integrando essas ferramentas com processos decisórios em um contexto de governança democrática ^48^ ^,^ ^49^ ^,^ ^50^.

A discussão acerca dos eixos de investigação, aqui abordados num campo conceitual, permite o entendimento de causalidades e a determinação dos impactos causados pela atividade humana, numa tentativa de estabelecer soluções equitativas e sustentáveis ^51^. Nessa corrente, destacam-se as características altamente poluidoras do processo produtivo do petróleo e dos seus derivados, bem como dos potenciais danos em diversos aspectos, como a cultura, que isoladamente impulsionam abordagens de estudos direcionados aos impactos ^52^.

No enfrentamento de desastres de derramamento de petróleo, que afetam aspectos físicos, sociais e econômicos das populações, a recuperação das comunidades requer esforços integrados para entender os riscos à saúde e à sustentabilidade comunitária. Defende-se, portanto, o uso de instrumentos que permitam a coleta sistemática e detalhada de dados, para uma compreensão completa dos impactos e da eficácia das ações de recuperação.

Na investigação sobre a explosão do Deepwater Horizon, constatou-se que dificuldades na contenção do desastre foram relacionadas a estimativas inadequadas de fluxos de óleo e à avaliação deficiente dos riscos da exposição e consumo de frutos do mar. A atividade pesqueira e a indústria de frutos do mar, essenciais para a economia local, foram significativamente afetadas pelos derramamentos. Além disso, há uma carência de informações sobre treinamento e especialização na avaliação de exposição ambiental dos profissionais envolvidos ^53^ ^,^ ^54^ ^,^ ^55^.

Observou-se uma carência de estudos que integrem a avaliação e o acompanhamento dos eventos, especialmente considerando questões sociais além da saúde. Essa lacuna pode ser atribuída ao paradigma biomédico, que tende a resistir a abordagens que incluem subjetividade, valores e aspectos simbólicos das relações sociais, focando principalmente na aplicação de instrumentos de investigação ^56^.

É importante ressaltar que, embora este estudo tenha se debruçado sobre a temática dos derramamentos de petróleo, outros eventos envolvendo produtos químicos, como agrotóxicos, são considerados à luz dessa discussão, pois esses também produzem impactos socioeconômicos e ambientais significativos, e de interesse para a saúde pública. Nesse diapasão, identificam-se dinâmicas de exposição combinada com consequências para os sistemas orgânicos dos indivíduos e sobre as estruturas socioeconômicas das áreas afetadas, também associados a mecanismos que produzem vulnerabilidade e injustiças num ciclo constante de degradação ^57^ ^,^ ^58^. Dessa forma, faz-se necessário considerar os riscos de acidentes químicos como questão de saúde pública.

O desenvolvimento industrial e a manipulação de produtos químicos apresentam potencial catastrófico que impulsiona a necessidade de políticas de planificação e prevenção à acidentes e desastres, com o controle emergencial e medidas de segurança para cada processo ^59^. Ademais, no contexto da exposição a substâncias tóxicas, é crucial o papel dos centros de informação toxicológica no manejo de incidentes graves enquanto os serviços de tratamento chegam às vítimas. Para promover políticas adequadas, é necessário adaptar essa ferramenta para garantir o fluxo eficiente de informações, notificações e encaminhamentos entre os subsistemas de diagnóstico, laboratórios, estatísticas, epidemiologia e vigilância comunitária ^60^.

A predominância de métodos observacionais (analíticos e descritivos) nos estudos selecionados reflete o interesse epidemiológico tradicional em estabelecer causalidade entre eventos, especialmente entre fatores contextuais e repercussões na saúde. Isso reforça a contribuição biomédica, ao mesmo tempo que ignora a importância de integrar aspectos sociais na construção do conhecimento científico ^61^.

Embora estudos observacionais sejam mais adequados para a avaliação da incidência de eventos com menor custo, é importante considerar a ocorrência de fatores de confusão entre os grupos observados durante a construção do desenho metodológico, sobretudo para adequada aplicação de instrumentos ^62^. Ainda, ressalta-se o seu menor grau de evidência e de recomendação quando comparados a estudos experimentais ou quase-experimentais, além da necessidade de se analisar a qualidade metodológica por diferentes estratégias, considerando as particularidades existentes nos estudos observacionais de coorte com grupo controle, sem grupo controle e do tipo transversais, como aqui apresentados ^32^ ^,^ ^63^.

Um ponto relevante é o distanciamento dos pesquisadores em relação aos objetos de estudo e às comunidades afetadas. Esse afastamento é evidenciado pela falta de pesquisas preliminares que orientem a elaboração metodológica e a escolha dos instrumentos de avaliação. A relação entre o método e o objeto é crucial para definir a abordagem científica, a ferramenta adequada e os resultados, garantindo uma visão integrada e concreta do problema ^64^.

Em outras palavras, a escolha dos instrumentos de avaliação para um método científico deve ser antecedida pela imersão e pela aproximação com a realidade vivenciada, a fim de compreendê-la e possibilitar a sua transformação; não apenas a sua análise crítica e fragmentada ^65^. Deste modo, para uma compreensão mais holística e precisa, e para melhor análise dos impactos, sugere-se um maior envolvimento das comunidades e a imersão dos pesquisadores nos territórios afetados.

A preferência por questionários nos estudos revisados parece ser justificada pela busca de informações diretamente dos pesquisados, permitindo inferências com menores custos, em menos tempo e com maior padronização. No entanto, há desafios como baixa taxa de retorno, exclusão de indivíduos com baixa escolaridade, respostas superficiais e inadequações ao contexto, que podem comprometer a representatividade da amostra e das análises ^66^.

Diante desse cenário, abordagens participativas e colaborativas, com o uso de instrumentos, podem fornecer uma visão mais detalhada dos impactos e das necessidades de populações afetadas. Elas superam as limitações dos métodos tradicionais, que frequentemente não envolvem suficientemente as comunidades afetadas, permitindo uma compreensão mais completa e compartilhada dos desastres ^53^.

A seleção de elementos binários nos estudos revisados permite que o respondente escolha entre duas opções, facilitando a reflexão antes da resposta. Em contraste, escalas do tipo Likert permitem variações de intensidade nas respostas, oferecendo uma análise mais detalhada. No entanto, escalas com mais pontos exigem maior análise e podem ser desafiadoras para respondentes com menor instrução ou maior carga cognitiva ^67^ ^,^ ^68^ ^,^ ^69^.

O uso de padrões livres de respostas em entrevistas favorece a interação entre o pesquisador e o participante, permitindo acesso a informações subjetivas com linguagem do senso comum e liberdade nas respostas ^70^. Ressalta-se, contudo, as limitações para esse tipo de abordagem instrumental, associadas a razões próprias dos indivíduos no fornecimento das respostas ou da influência exercida pelo aspecto pessoal do aplicador, dentre outras desvantagens que comprometem o recorte da realidade analisada ^70^.

## Considerações finais

Os artigos revisados utilizaram diferentes instrumentos para avaliar a exposição aos derramamentos, os impactos na saúde (principalmente mentais e psicológicos), e a percepção de riscos e danos ambientais, além dos efeitos socioeconômicos e sobre a coesão social. Embora esses instrumentos ofereçam uma visão ampliada dos eventos, há uma falta de estudos que abordem de forma integrada a dimensão ambiental e socioeconômica dos impactos. Futuras pesquisas devem expandir a análise sobre a consistência, a confiabilidade e as adaptações transculturais dos instrumentos. Essas informações podem ajudar a criar uma matriz multidimensional para monitorar desastres, facilitando decisões rápidas e eficazes na elaboração de políticas de proteção e recuperação.
